# Supplementary material for: CDK1-loaded extracellular vesicles promote cell cycle to reverse impaired wound healing in diabetic obese mice
Source: Mol Ther. 2025 Jan 25;33(3):1118–33. doi: 10.1016/j.ymthe.2025.01.039 (PMC11897770; doi:10.1016/j.ymthe.2025.01.039)
Supplement: Document S1. Figures S1–S6 and Table S1 [file mmc1.pdf]

YMTHE, Volume *33*

Supplemental Information

CDK1-loaded extracellular vesicles promote cell cycle to reverse impaired wound healing in diabetic obese mice

Wooil Choi, Dong Jun Park, Robert A. Dorschner, Keita Nakatsutsumi, Michelle Yi, and Brian P. Eliceiri

Supplemental material

Table S1. List of materials

REAGENT or RESOURCE	SOURCE	IDENTIFIER
Antibodies		
anti-Alix mAb	Cell Signaling Technology	Cat# 92880; RRID: AB_2800192
anti-Calnexin mAb	Cell Signaling Technology	Cat# 2679; RRID: AB_2228381
anti-CD63 pAb	Thermo Fisher	Cat# PA5-92370; RRID: AB_2806456
anti-Cdc2 mAb	Cell Signaling Technology	Cat# 9116; RRID: AB_2074795
anti-CD81 mAb	Cell Signaling Technology	Cat# 56039; RRID: AB_2924772
anti-Ki-67 mAb	Thermo Fisher	Cat# MA5-14520; RRID: AB_10979488
anti-Akt mAb	Cell Signaling Technology	Cat# 4691; RRID: AB_915783
anti-pAkt (Ser473) mAb	Cell Signaling Technology	Cat# 4060; RRID: AB_2315049
anti-4E-BP1 mAb	Cell Signaling Technology	Cat# 9644; RRID: AB_2097841
anti-p4E-BP1 (Thr37/46) mAb	Cell Signaling Technology	Cat# 2855; RRID: AB_560835
anti-Erk1/2 mAb	Cell Signaling Technology	Cat# 4695; RRID: AB_390779
anti-pErk1/2 (Thr202/Thr204) mAb	Cell Signaling Technology	Cat# 4370; RRID: AB_2315112
anti-p27 Kip1 mAb	Cell Signaling Technology	Cat# 3686; RRID: AB_2077850
anti-pHistone H3 (Ser10) mAb	Cell Signaling Technology	Cat# 9706; RRID: AB_331748
anti-Acetyl-α-tubulin mAb	Cell Signaling Technology	Cat# 5335; RRID: AB_10544694
anti-β-actin mAb	Cell Signlaing Technology	Cat# 3700; RRID: AB_2242334
anti-Mouse IgG, HRP-linked	Cell Signaling Technology	Cat# 7076; RRID: AB_330924
anti-Rabbit IgG, HRP-linked	Cell Signaling Technology	Cat# 7074; RRID: AB_2099233
anti-Rabbit IgG-Alexa Fluor^{TM} 488	Thermo Fisher	Cat# A11008; RRID: AB_143165
anti-Rabbit IgG-Alexa Fluor^{TM} 546	Thermo Fisher	Cat# A11010; RRID: AB_2534077
anti-Mouse IgG-Alexa Fluor^{TM} 488	Thermo Fisher	Cat# A11029; RRID: AB_2534088
anti-Mouse IgG-Alexa Fluor^{TM} 546	Thermo Fisher	Cat# A11030; RRID: AB_2737024
anti-hCD9 Ab	Cellarcus Bioscience Inc.	Cat# CBS10-PE
anti-hCD63 Ab	Cellarcus Bioscience Inc.	Cat# CBS11-PE
anti-hCD81 Ab	Cellarcus Bioscience Inc.	Cat# CBS12-PE
Isotype IgG1, κ control	Cellarcus Bioscience Inc.	Cat# CBS29-PE
Chemicals, Enzymes		
DMEM-high glucose medium	Thermo Fisher	Cat# 12430054
Fetal Bovine Serum (FBS)	Sigma Aldrich	Cat# F0926
Antibiotic-Antimycotic (100X)	Thermo Fisher	Cat# 15240062
0.4% Trypan blue	Thermo Fisher	Cat# T10282
Xho I	New England Biolabs	Cat# R0146
Not I	New England Biolabs	Cat# R1089S
Lipofectamine^{TM} 2000	Thermo Fisher	Cat# L3000150

Opti-MEM™	Thermo Fisher	Cat# 31985062
Bovine exosome-delpleted FBS	System Biosciences	Cat# EXO-FBS-50A-1
ExoQuick-TC	System Biosciences	Cat# EXOTC-50A-1
Pierce™ RIPA buffer	Thermo Fisher	Cat# 89901
Halt™ Protease & phosphatase inhibitor cocktail	Thermo Fisher	Cat# 78442
Pierce™ Bovine serum albumin	Thermo Fisher	Cat# 23209
NuPAGE™ LDS sample buffer (4X)	Thermo Fisher	Cat# NP008
Dithiothreitol (DTT)	Thermo Fisher	Cat# 15508013
Non-fat dry milk	Cell Signaling Technology	Cat# 9999
SignalFire™ Elie ECL reagent	Cell Signaling Technology	Cat# 12757
vCal nanoRainbow beads	Cellarcus Bioscience Inc.	Cat# CBS6
Mitomycin C	Sigma-Aldrich	Cat# M0440
Tissue-Tek® O.C.T. Compound	Sakura	Cat# 4583
Paraformaldehyde aqueous solution	Electron Microscopy Science	Cat# 15710
Triton X 100		
Fluoroshield™ with DAPI	Sigma-Aldrich	Cat# F6057
LysoTracker™ Red DND-99	Thermo Fisher	Cat# L7528
1X Phosphate-buffered saline	Thermo Fisher	Cat# 10010023
Tris-buffered saline with Tween 20	Cell Signaling Technology	Cat# 9997
MACSQuant running buffer	Miltenyi Biotec	Cat# 130-092-747
Critical Commercial Assay		
ZymoPURE II plasmid maxiprep kit	Zymo Research	Cat# D4203
Pierce™ BCA protein assay	Thermo Fisher	Cat# 23225
Pierce™ Reversibl protein stain kit	Thermo Fisher	Cat# 24585
vFC™ Vesicle flow cytometry assay	Cellarcus Biosciences Inc.	Cat# CBS4HP
Cell counting kit-8 assay	Dojindo	Cat# CK04
Cell cycle assay solution deep red	Dojindo	Cat# C548
Recombinant DNA		
XPack CMV-XP-MCS	System Biosciences	Cat# XPAK510PA-1
XPack CMV-XP-GFP	System Biosciences	Cat# XPAK530CL-1
Human CDK1 untagged clone	Origene	Cat# SC111605
Experimental models: Animals		
B6.VJS(D)-Lepr$^{db/db}$/J mice	Jackson lab	JAX000697; RRID: IMSR_JAX:000697
Experimental models: Cell lines		
HEK293T	Takara Bio	Cat# 632180; RRID: CVCL_4401
HaCaT		
Experimental models: Bacterial strain		
NEB stable competent *E. coli* (K12)	New England Biolabs	Cat# C3040I

Software and Equipment		
GraphPad Prism10 software	GraphPad Software	https://www.graphpad.com/
FCS express	De Novo	https://denovosoftware.com/
Aperio ImageScope (v12.4.6)	Leica Biosystem	https://www.leicabiosystems.com/digital-pathology/manage/aperio-imagescope/
ZEN blue (v3.4.91)	Carl Zeiss Microscopy GmbH	https://www.zeiss.com/microscopy/en/products/software/zeiss-zen.html
FlowJo software (v10.8.2)	FlowJo™, LLC	https://www.flowjo.com/
Image J (v1.54i)	NIH	https://imagej.nih.gov/ij/download.html
CytoFLEX Flow Cytometer	Beckman Coulter	https://www.beckman.com/flow-cytometry/research-flow-cytometers/cytoflex
Xenogen IVIS-Lumina	Caliper life science Inc.	http://www.caliperls.com
MACSQuant Analyzer 10	Miltenyi Biotec	https://www.miltenyibiotec.com/US-en/products/macsquant-analyzer-10.html
Retiga R6 CCD camera	Teledyne photometrics	https://www.photometrics.com/products/qimaging-ccd-family/retiga-r6
AXR confocal microscopy	Nikon	http://www.microscope.healthcare.nikon.com
Others		
12% Bis-tris mini gel	Thermo Fisher	Cat# NP0342BOX
PVDF membrane	Thermo Fisher	Cat# LC2005
96-well v bottom plate	Sarstedt	Cat# NC0068972
Oasis Nylon Suture	Med Vet International	Cat# MV-662
Betadine (10% povidone-iodine solution)	Thermo Fisher	Cat# NDC 67618-150-01
4 mm punch	Acuderm Inc.	Cat# P450
Silicone ring	Crace Bio-Labs	Cat# GBLRD476687
Surgical glue	Thermo Fisher	Cat# 1469SB
Tegaderm™ transparent dressing film	3M	Cat# 1622w
2-well silicon insert	Ibidi	Cat# 80209
Black 12-well plate with glass like bottom	Cellvis	Cat# P12-1.5P

Figure S1

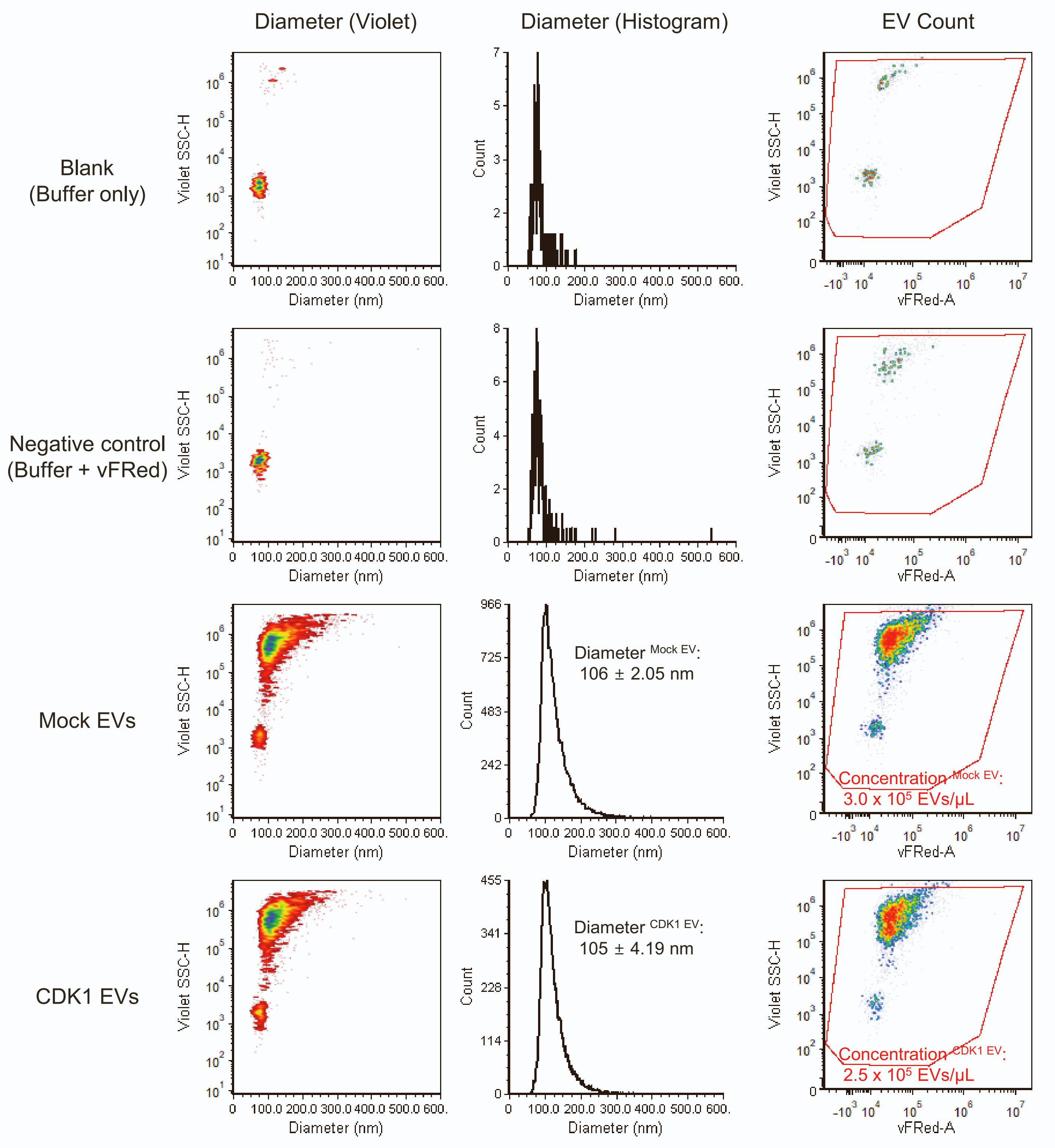

Figure S1. Controls for single vesicle flow cytometry (vFC) based on concentration of samples by polymeric precipitation. Representative controls showing event distribution in a running buffer only (Blank), negative control that has running buffer with fluorescent lipophilic membrane dye vFRed without sample, Mock sEVs and CDK1-loaded sEVs with vFRed dye. The left column contains plots of size based on calibration with Nanorainbow beads vs Violet SSC-H. The middle column shows diameter vs. count to obtain a size distribution and further supporting the ~110 nm population. The right column is a distribution of vFRed positive events vs. Violet SSC-H with concentration calculated based on events, rate, and volume.

Figure S2

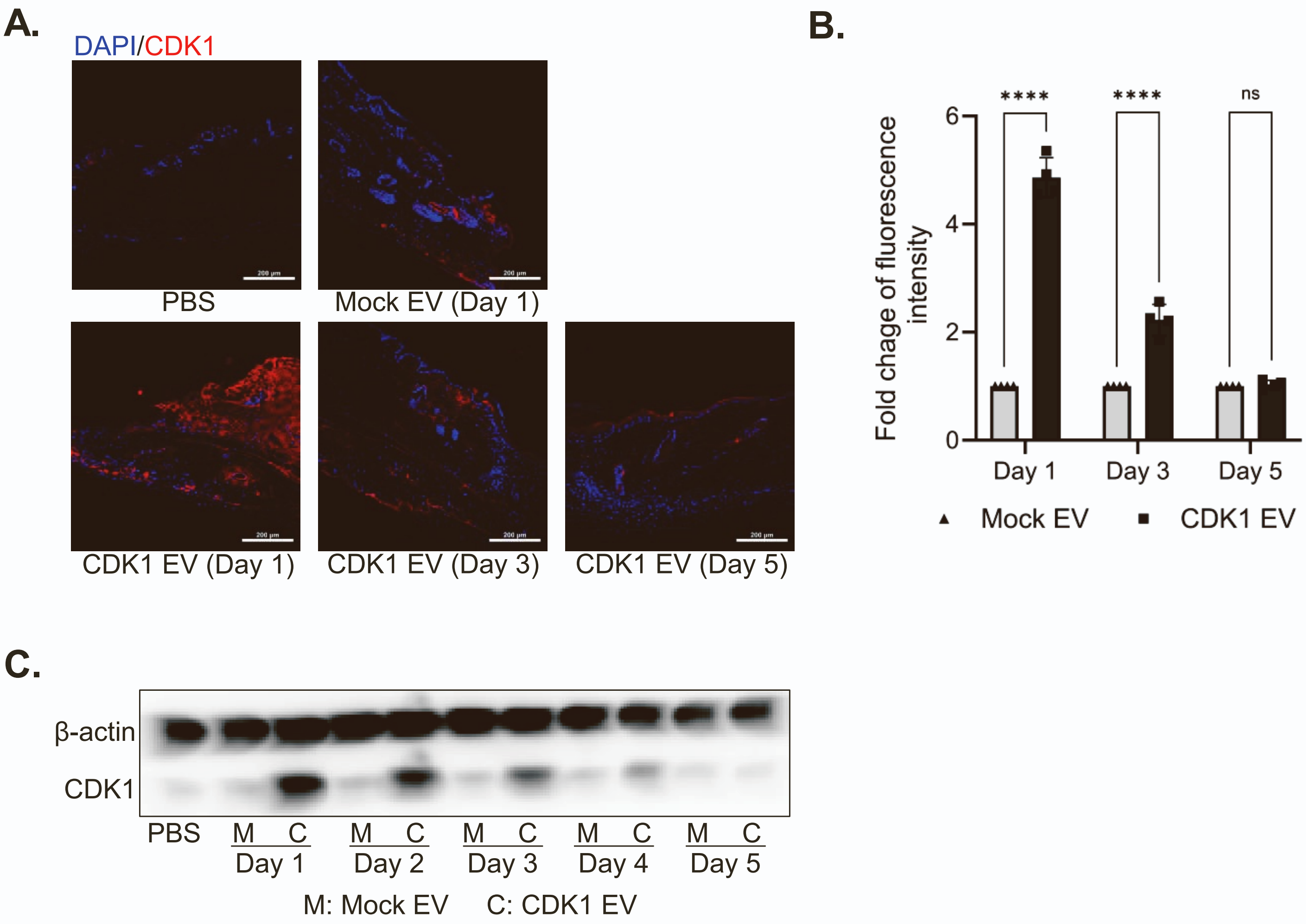

Figure S2. Retention time of CDK1-loaded sEVs in wound area. (A) Representative immunofluorescent images of wound margin with an anti-CDK1 antibody (red), and counter-stained with a nuclear stain (blue) post-sEV treatment (Scale bar: 200 μm) and (B) their quantification (n = 4, $^{****}p < 0.0001$). (C) Immunoblotting of CDK1 in wound area.

Figure S3

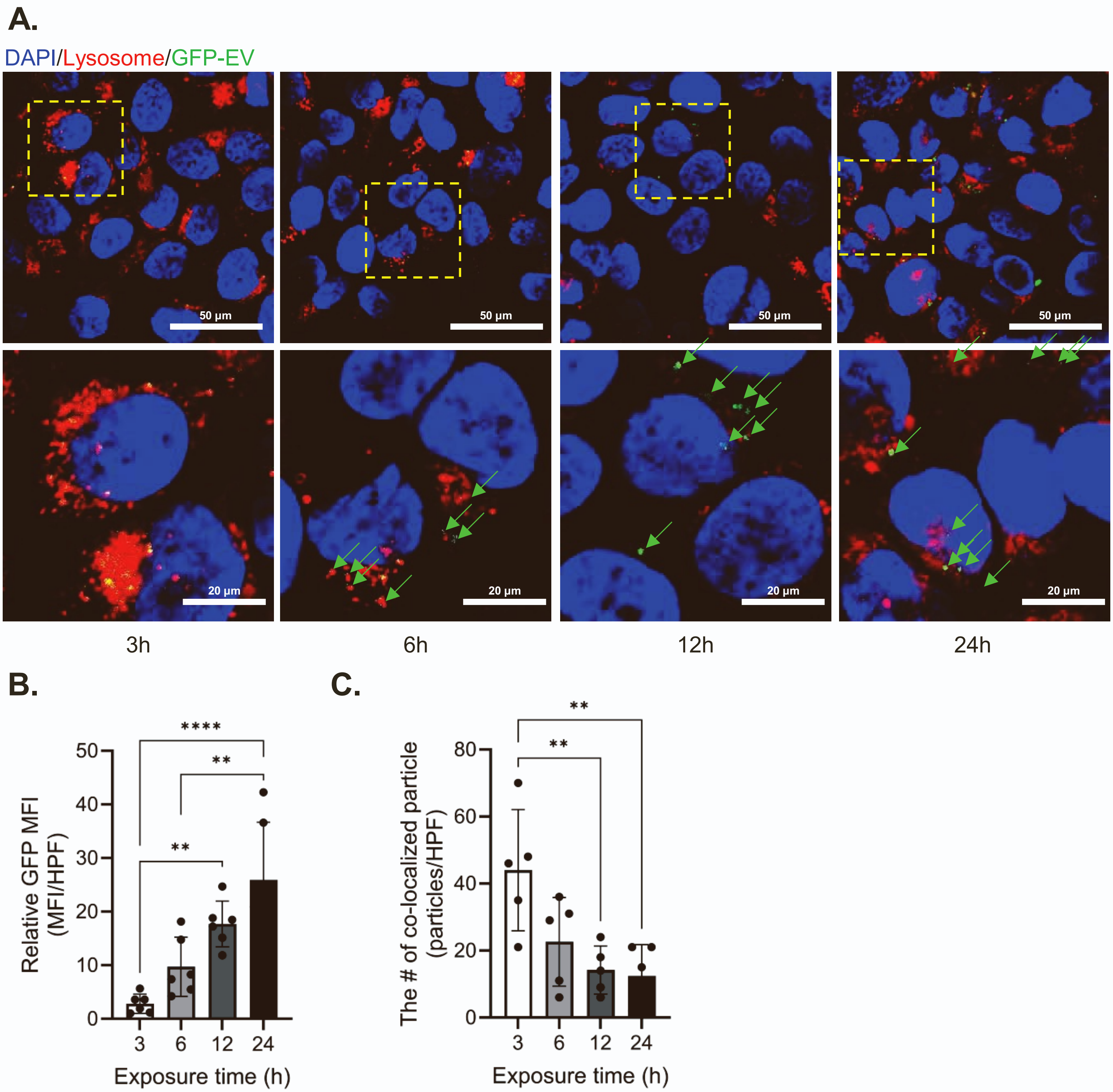

Figure S3. EV cargo release occurs from lysosomes. (A) Immunofluorescent images stained with lysotracker (red) incubated with GFP-loaded sEVs (green) for 3, 6, 12, and 24 hours. Yellow particles represent co-localization of lysosome and GFP and green arrows point escaped GFP (Scale bar: 50 µm). (B) quantified fluorescence intensity of GFP for 24 hours ($n = 6$, $^{****}p < 0.0001$, $^{**}p < 0.01$). (C) quantification of co-localization of lysosome and GFP ($n = 6$, $^{**}p < 0.01$).

Figure S4

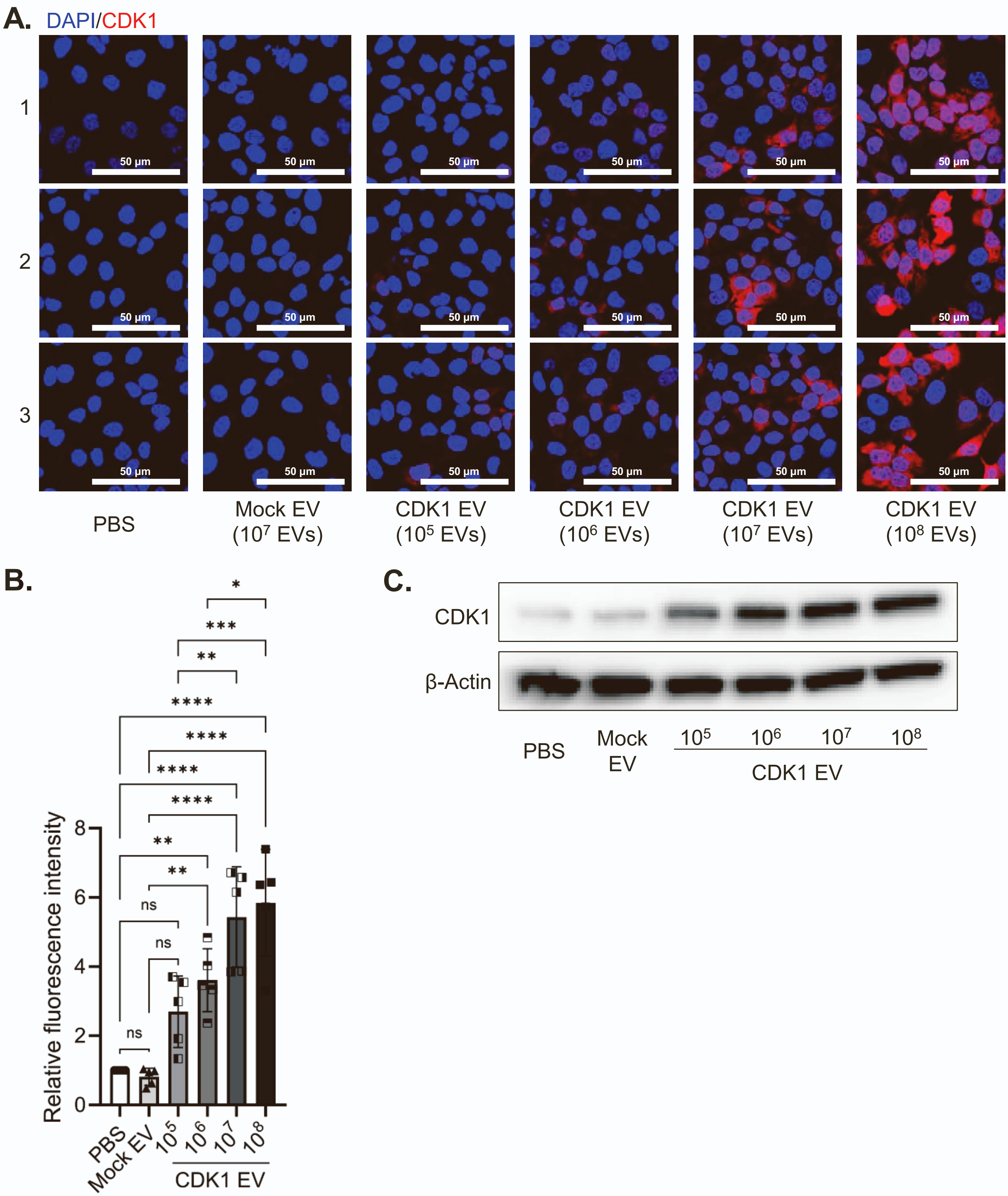

Figure S4. Release kinetics of CDK1 in recipient keratinocytes from sEVs. (A) immunofluorescent images stained with CDK1 (red), and counter stained with DAPI for nuclei (blue) after CDK1-loaded sEV treatment (Scale bar: 50 µm), and their quantification (B), (n = 5, $^{****}p < 0.0001$, $^{***}p < 0.001$, $^{**}p < 0.01$, $^{*}p < 0.05$). (C) immunoblot of CDK1 in recipient whole cell lysate after CDK1-loaded sEV treatment.

Figure S5

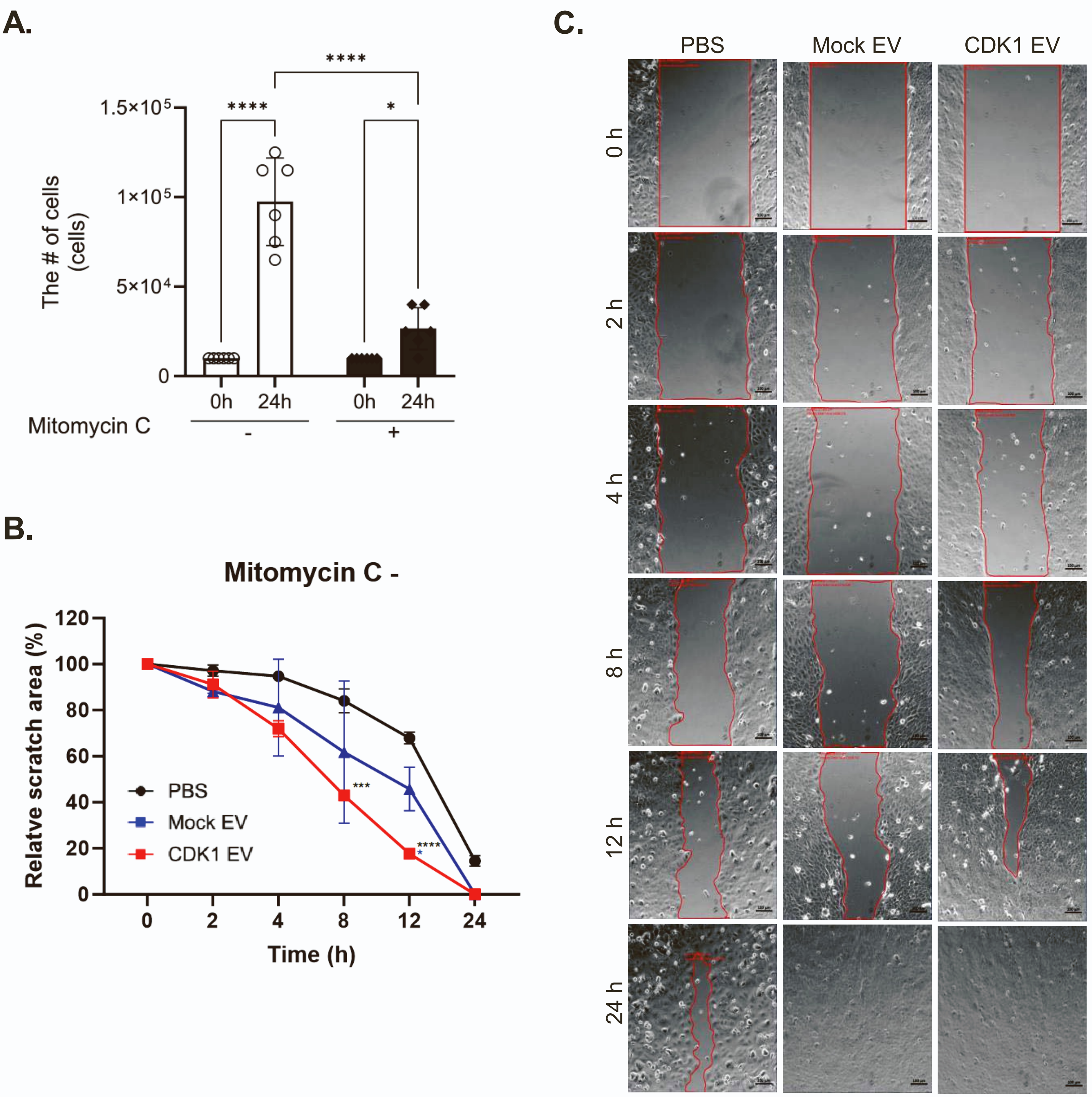

Figure S5. In vitro migration assay by CDK1 delivered to human keratinocyte by sEV treatment. (A) Proliferation inhibitory efficacy of mitomycin C in human keratinocyte (n = 6, $^{****}p < 0.0001$). (B) quantification of cell migration without proliferation arrest by mitomycin C, and their representative images (C), (Scale bar: 100 μm, $^{****}p < 0.0001$, $^{***}p < 0.001$, $^{*}p < 0.05$).

Figure S6

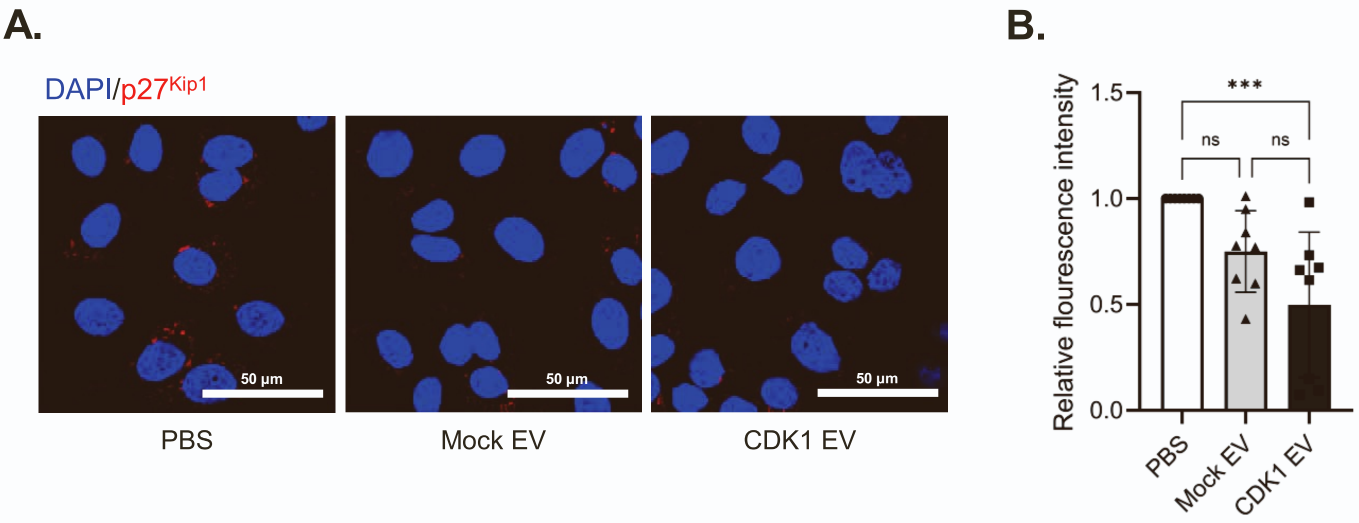

Figure S6. Repression of p27^{Kip1} by activated AKT and ERK signaling. (A) immunofluorescent images stained with p27^{Kip1} (red), and counter stained with DAPI for nuclei (blue) after CDK1-loaded sEV treatment, and their quantification (B), (Scale bar: 50 µm, n = 8, $^{***}p < 0.001$).
