# Supplementary material for: CDK1-loaded extracellular vesicles promote cell cycle to reverse impaired wound healing in diabetic obese mice
Source: Mol Ther. 2025 Jan 25;33(3):1118–33. doi: 10.1016/j.ymthe.2025.01.039 (PMC11897770; doi:10.1016/j.ymthe.2025.01.039)
Supplement: Document S2. Article plus supplemental information [file mmc2.pdf]

Molecular Therapy
Original Article

CDK1-loaded extracellular vesicles promote cell cycle to reverse impaired wound healing in diabetic obese mice

Wooil Choi,[1] Dong Jun Park,[1] Robert A. Dorschner,[2] Keita Nakatsutsumi,[1] Michelle Yi,[1] and Brian P. Eliceiri[1,2]

[1]Department of Surgery, University of California, San Diego, La Jolla, CA 92093, USA; [2]Department of Dermatology, University of California, San Diego, La Jolla, CA 92093, USA

Small extracellular vesicles (sEVs) mediate intercellular signaling to coordinate the proliferation of cell types that promote re-epithelialization of skin following injury. Cyclin-dependent kinase 1 (CDK1) drives cell division and is a key regulator of entry to the cell cycle. To understand the potential of sEV-mediated delivery of CDK1 to reverse impaired wound healing, we generated CDK1-loaded sEVs (CDK1-sEVs) and evaluated their ability to mediate cell proliferation, re-epithelialization, and downstream signaling responses in the wound bed. We found that treatment of human keratinocytes with CDK1-sEVs increased phosphorylation of the CDK1 target, eukaryotic translation inhibition factor 4E-binding protein 1 (4E-BP1), and histone H3 within 24 h via AKT and ERK phosphorylation, driving increased proliferation and cell migration. Treatment of the wound bed of diabetic obese mice, a model of delayed wound healing, with a single dose of CDK1-sEVs accelerated wound closure, increased re-epithelialization, and promoted the proliferation of keratinocytes. These studies show that delivery of CDK1 by sEVs can stimulate selective and transient proliferation of cell types that increase re-epithelialization and promote proliferation of keratinocytes to accelerate wound healing.

INTRODUCTION

Cutaneous wound healing is a complex tissue repair process comprising stages of inflammation and proliferation, followed by remodeling. This process is impaired in diabetic patients.[1] Up to 34% of diabetic patients experience impaired wound healing in their lifetime[2] with chronic wounds that are characterized by dysregulated inflammation, decreased angiogenesis, and disrupted keratinocyte migration.[3–6] Recent work from our lab and others has focused on the identification of nucleic acid or protein payloads of pro-reparative extracellular vesicles (EVs) in preclinical models.[7,8] Although the impaired wound-healing phenotype in diabetes is multi-factorial, recent studies implicate the disruption of cell-cycle mediators, which are important molecular switches to control entry into the cell cycle. Of the many cyclin-dependent kinases (CDKs) studied to date, only CDK1 is known to be indispensable for the regulation of entry into the cell cycle,[9,10] and it has been shown to be downregulated in the wound site of diabetic patients. This is in contrast to its complex partner, cyclins.[11] This impaired capacity for cell-cycle progression underlies the reduced proliferation and migration of resident stem cells that is characteristic of the diabetic wound. In preclinical models, the loss of CDK1 reduces cell proliferation and lipid metabolism, which results in insulin resistance.[12] Although a role for CDK1 as a regulator of cutaneous wound healing was supported by the reduced kinetics of wound closure in CDK1 knockout mice, the specific mechanism remained unclear. In this study, we identify a mechanism for CDK1 regulation of wound healing and demonstrate the potential for CDK1-based therapeutics by loading small EVs (sEVs) with CDK1 protein to deliver stable, biologically active, pro-reparative EVs that modulate defined molecular endpoints relevant to CDK1 action in the cytoplasm and nucleus and accelerate wound healing.

Naturally occurring EVs promote epidermal homeostasis[13] based on the intercellular exchange of biochemically active payloads, such as nucleic acids, proteins, and lipids.[14,15] Previous studies have identified specific pro-reparative EV payloads that stimulate angiogenesis, timely resolution of inflammation, and proliferation of specific cell types in the skin.[16,17] For example, functional studies of specific microRNAs (miRNAs)[18–20] or circular RNAs,[21] either through passive or active EV loading, have shown beneficial effects on wound closure, including promoting cell proliferation, migration, and signaling.[22] However, the efficacy of RNA-loaded EVs in promoting wound healing is limited because of their relatively low abundance based on stoichiometry studies, RNA instability, and the underappreciated importance of active loading for therapeutic testing.[23,24] To address some of these limitations, we and others have developed and validated specific protein-loaded EV payloads to identify pro-reparative properties of specific proteins in specific subcellular compartments.[25] For example, interleukin 10-loaded EVs ameliorate kidney injury via the mammalian target of rapamycin (mTOR) pathway,[26] delivery of IκB-loaded EVs reduce lipopolysaccharide-induced inflammation in development,[27] and catalase-loaded EVs protect neurons against oxidative

Received 26 August 2024; accepted 22 January 2025;
https://doi.org/10.1016/j.ymthe.2025.01.039.

Correspondence: Brian P. Eliceiri, Department of Surgery, University of California, San Diego, La Jolla, CA 92093, USA.
E-mail: beliceiri@health.ucsd.edu

 © 2025 The Authors.
Published by Elsevier Inc. on behalf of The American Society of Gene and Cell Therapy.
This is an open access article under the CC BY license (http://creativecommons.org/licenses/by/4.0/).

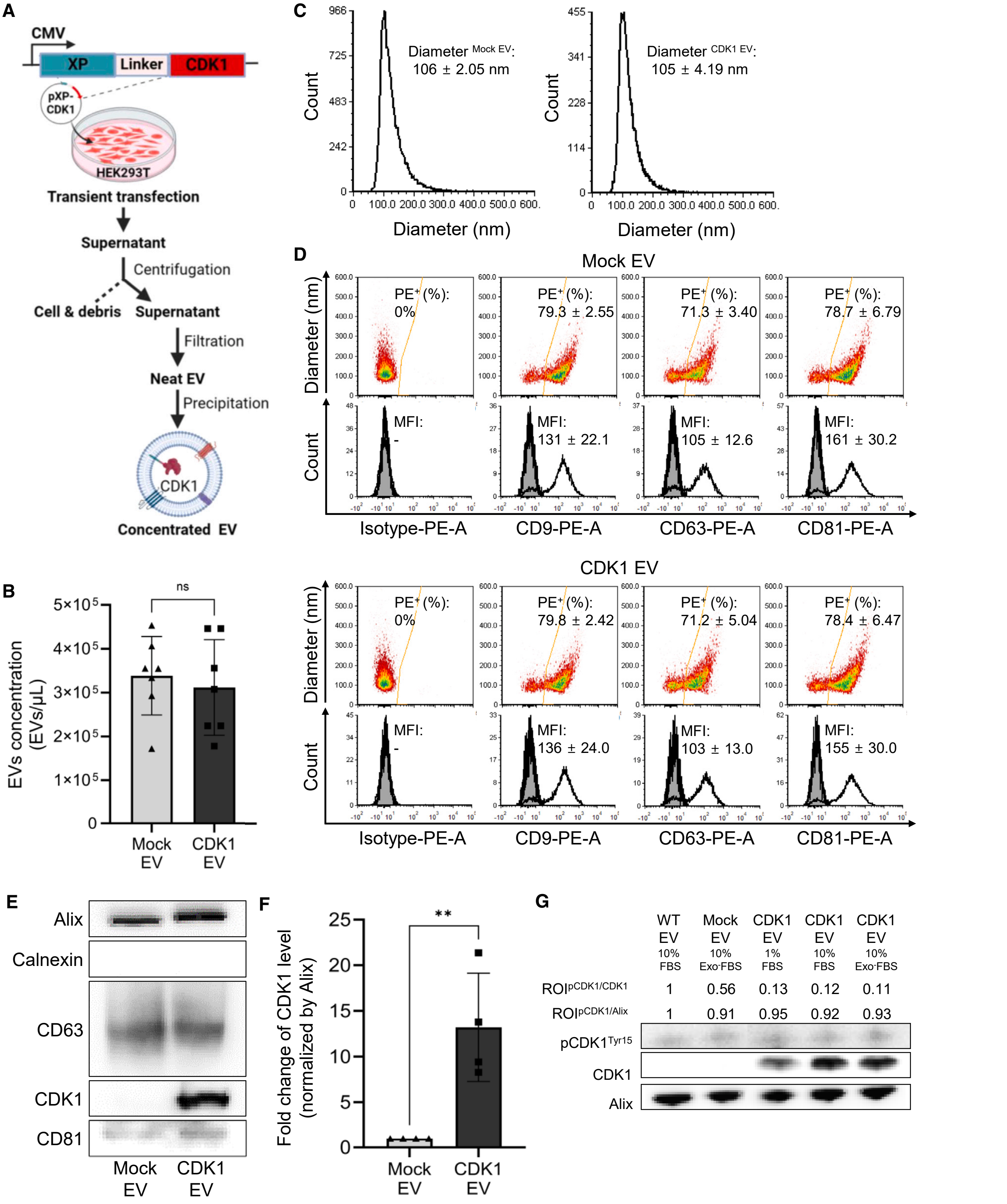

(legend on next page)

stress.[28] We have shown that a subset of serine protease inhibitors (serpins) expressed as fusion proteins with specific membrane targeting domains (i.e., myristoylation) can be actively loaded into EVs.[29] Using this myristoylation fusion approach, membrane-targeted serpin-loaded EVs were released into conditioned media from EV donor cells, demonstrating loading of a specific protein payload into the EVs, which was subsequently released into recipient cells. For example, EVs released from cultured human donor cells were purified from conditioned media and used to treat recipient cells and promote the migration of human keratinocytes. These human-derived EVs, including HEK293T cell-derived EVs, show no toxicity and immunogenicity with or without genetic engineering.[30,31] Similarly, EVs released from mouse donor cells engineered to express actively loaded serpin-released EVs rescued the impaired wound-healing phenotype in diabetic obese mice.[19] Here, we used this established EV loading strategy to test the activity of CDK1-loaded EVs on wound closure and determine whether specific molecular endpoints of CDK1 action could be observed in recipient cells.

CDKs are key regulators of the cell cycle,[32] with CDK1 targeting specific cytosolic signaling intermediates such as 3-phosphoinositide-dependent protein kinase-1 that stimulate the phosphorylation of alpha serine/threonine-protein kinase (AKT) and one of its effectors, extracellular signal-regulated kinase (ERK).[33,34] AKT phosphorylation suppresses CDK1 inhibitors like p27kip1,[35,36] while ERK acts upon the eukaryotic translation initiation factor 4E-binding protein 1 (4E-BP1).[37,38] Therefore, we assessed the activation of 4E-BP1 based on its cytosolic phosphorylation and its association with increased proliferation,[39–43] as well as histone H3, another CDK1 effector that is phosphorylated and directly associated with chromatin remodeling including condensation during mitosis.[44,45] We show here that treatment of cells with CDK1-loaded sEVs promoted the localization of phosphorylated histone H3, consistent with the hypothesis that delivery of CDK1 protein induced histone H3 phosphorylation,[46] which promoted entry into mitosis.

In this study, we engineered actively loaded CDK1 protein payload in sEVs, which accelerated wound closure and cell proliferation in full-thickness murine wounds. Treatment of cultured cells with CDK1-loaded sEVs stimulated human keratinocyte migration and proliferation and signaling of AKT, ERK, and 4E-BP. CDK1-loaded sEVs induced entry into the cell cycle that was associated with increased levels of phosphorylated histone H3 in the nucleus. For future directions, we expect that engineering of target-specific EVs through surface modification could enhance drug delivery of well-defined payloads such as CDK1.[47,48] These findings show that CDK1-loaded sEVs have a pro-reparative biological activity in accelerating wound closure in relevant preclinical models of impaired wound healing,[29] demonstrating the therapeutic potential of this approach.

RESULTS

Engineering and validation of CDK1-loaded sEVs

To generate CDK1-loaded sEVs, we generated a fusion protein of CDK1 with an N-terminal myristoylation sequence (myr-CDK1),[49,50] which we have previously demonstrated efficiently loads sEVs with protein cargoes[29] (Figure 1A). sEVs were purified from the conditioned media of cells transfected with myr-CDK1 or control, as previously described. Purified EVs were characterized[51] via vesicle flow cytometry (vFC)[52] (Figure S1), demonstrating similar sEV concentrations (Figure 1B), sizes (Figure 1C), and surface expression of the canonical sEV tetraspanin proteins CD9, CD63, and CD81 (Figure 1D) in both myr-CDK1 and mock-transfected cell sEVs. Immunoblotting showed an increased expression of CDK1 in CDK1-loaded sEVs compared to mock sEVs, while similar levels of Alix, CD63, and CD81 were observed on both populations of sEVs, and an absence of the endoplasmic reticulum protein Calnexin (Figure 1E). Quantification of CDK1 protein was normalized to Alix (Figure 1F) to establish engineered CDK1-loaded sEVs for further activity testing. Given that Massacci et al. demonstrated that CDK1 is active when inhibitory residues such as Tyr15 appear hypo-phosphorylated,[53] we confirmed the phosphorylation status of CDK1 loaded into sEVs by immunoblot. We observed that overexpression of CDK1 and loading into sEVs did not affect CDK1 phosphorylation, and the amount of $pCDK1^{Tyr15}$ in sEVs was similar to that of the wild-type and mock sEVs (Figure 1G).

Testing the activity of CDK1-loaded sEVs in an animal model of impaired wound healing

To determine the wound-healing activity of CDK1-loaded sEVs *in vivo*, we used 12- to 16-week-old leptin receptor knockout mice, an established and well-defined model of impaired wound healing that is the result of delayed epithelialization related to hyperglycemia and obesity,[19,29,54] and EVs loaded with human CDK1, which shows >97% homology to mouse CDK1. To examine the effect of CDK1 in the diabetic wound model immediately after wound formation, purified sEVs were added as a single topical treatment to a splinted full-thickness excisional wound on the dorsum of the mouse, and the wound diameter imaged over a 9-day time course (Figure 2A). We observed that wounds treated with CDK1-loaded sEVs demonstrated accelerated wound closure kinetics compared to wounds treated with mock sEVs (Figures 2B and 2C). Wound treatment with either CDK1-loaded sEVs or mock sEVs increased wound closure compared to PBS treatment, as previously shown.[19,29] At days 3 and 5, we observed a statistically significant improvement in the pro-reparative activity of CDK1-loaded sEVs vs. mock sEVs. The

Figure 1. Characterization of CDK1-loaded sEVs

(A) Schematic of engineering CDK1-loaded sEVs. (B) Determination of EV concentration of mock (empty vector) and CDK1-loaded sEVs (n = 7 each). (C) Size distribution of sEVs (n = 7). (D) Representative vFC analyses of sEVs (n = 7). MFI, mean fluorescent intensity. (E) Immunoblotting of sEVs using EV markers, CDK1 and calnexin. (F) Normalized CDK1 expression based on immunoblotting, region of interest values shown (n = 4; $^{**}p < 0.01$). (G) Immunoblotting of sEVs using phosphorylation of $CDK1^{Tyr15}$ vs. pan-CDK1, and Alix as EV marker.

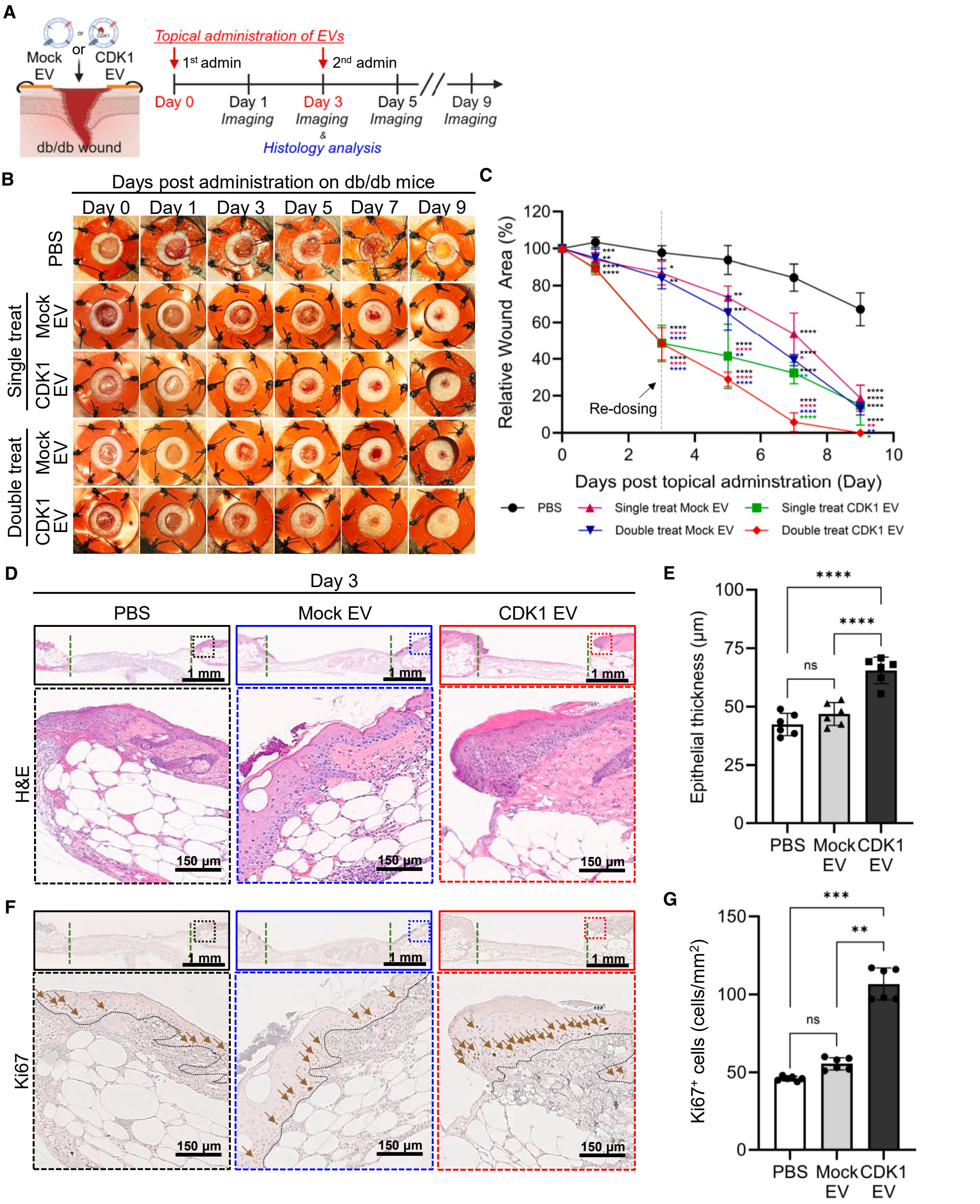

(legend on next page)

extent of the wound-healing response was similar between CDK1 and mock sEVs by day 9, suggesting that CDK1 acts in the early phase of tissue repair.

Immunofluorescent imaging and immunoblotting analysis indicated CDK1 remained significantly elevated for up to 3 days after delivery by sEV, and declined by day 5 after delivery (Figures S2A–S2C). To test the pro-reparative activity of multiple doses of CDK1 sEVs, we administered CDK1 sEVs on days 0 and 3 (double-treated group; CDK1-loaded sEVs vs. mock sEVs). The double-treated CDK1-loaded sEV double-treated mice showed a significant improvement in wound closure in comparison to mice treated with a single dose of CDK1-loaded sEV or mice treated with either one or two doses of mock EVs.

Analysis of tissue histology showed increased epithelial thickness and extension of migratory epithelium (epithelial tongue)[55] (Figures 2D and 2E), which was associated with increased cell proliferation determined by Ki67 immunostaining (Figures 2F, 2G, and S2D). These results supported a model that delivery of CDK1-loaded sEVs enhances tissue repair and promotes re-epithelialization via increased cell proliferation *in vivo*.

Testing biochemical activity of CDK1 delivered by sEVs to human keratinocyte

To test the biochemical activity of CDK1-loaded sEVs on a human keratinocyte cell line (HaCaT), we immunostained sEV-treated cells with an anti-CDK1 antibody to determine the intracellular distribution. We observed that CDK1 protein payloads were present in the cytosol of cells treated with CDK1-sEVs (Figure 3A) and distinct from the lysosomal distribution that is often associated with sEV uptake in recipient cells[56] (Figure S3A). This is consistent with the clathrin-mediated endocytosis and micropinocytosis being the primary mechanism of EV uptake.[57] To further investigate protein uptake and release in recipient cells, we generated EVs that were engineered to express green fluorescent protein (GFP) using the same myristoylation tag approach as the engineering of CDK1-loaded sEVs, and we observed the accumulation of GFP-loaded sEVs over a time course of 6–24 h, with increased uptake by 24 h (Figure S3B). By quantifying the association of GFP-loaded sEVs with LysoTracker, a lysosome marker, we demonstrated that GFP-loaded sEVs escape into the cytosol within 6 h (Figure S3C). The release of the engineered sEVs into the cytosol was further investigated by serial dilution showing an optimal dosing of 10^{7}–10^{8} sEVs/100 μL to 10^{5} cells (Figures S4A–S4C). These data demonstrate that GFP-loaded sEVs were taken up by recipient cells, undergo endosomal escape, and have the potential to exert biochemical activity in the cytosol.

To determine the mechanism for the improvement in wound healing, we assessed the effects of CDK1-loaded sEVs on cell proliferation and cell migration. Cell proliferation was assessed by the increase in the number of cells 24 h after sEV treatment relative to the increase in the number of cells 24 h after treatment with PBS. We observed a 25.6% increase following treatment with CDK1 sEVs compared to mock sEVs (Figures 3B and 3C). To determine whether the CDK1-loaded sEVs might regulate migration in addition to proliferation to enhance wound healing, we used a modified scratch assay in which the migration of HaCaT keratinocytes was monitored in the presence or absence of mitomycin C (Figure S5A). We observed that in the presence of mitomycin C, the treatment of recipient cells with CDK1-loaded sEVs increased migration, even in the absence of proliferation, with accelerated scratch closure (Figures 3C and 3D). In the absence of mitomycin C, we observed even more rapid gap closure in this model (Figures S5B and S5C). To determine whether the treatment of cells with CDK1-loaded sEVs affected the cell cycle, we stained cells to measure DNA content (Figure 3E) and observed a consistent CDK1-mediated entry into G2/M phase based on the increase in DNA content (Figure 3F). These findings supported a model that delivery of CDK1-loaded sEVs promoted entry in the cell cycle that was associated with increased keratinocyte migration and proliferation.

Signaling pathway by exosomal CDK1 in cytoplasm

We next evaluated the effect of CDK1-loaded sEV treatment on known downstream molecular endpoints of CDK1. 4E-BP1 phosphorylation is associated with the phosphatidylinositol 3-kinase (PI3K)/AKT signaling pathway, which integrates both intracellular and extracellular signals to regulate cell metabolism, growth, and proliferation.[58,59] Because CDK1 regulates 4E-BP1$^{Thr202/Try204}$ through the activation of AKT, we focused on CDK1-mediated phosphorylation changes in AKT^{Ser473} and ERK$^{Thr37/46}$ as key downstream mediators. We observed that treatment of cells with CDK1-loaded sEVs increased the phosphorylation of AKT^{Ser473} (2.0-fold; Figures 4A and 4B), ERK$^{Thr202/Tyr204}$ (7.4-fold; Figures 4C and 4D), and 4E-BP1$^{Thr37/46}$ (6.0-fold; Figures 4E and 4F) relative to mock EV-treated cells. In addition, activation of AKT and ERK signaling suppresses the p27^{Kip1} through the various downstream pathways.[60–62] After CDK1-loaded sEV treatment, we also identified suppressed p27^{Kip1} expression by AKT and ERK phosphorylation promoting the cell cycle (Figures S6A and S6B). We then examined the effect of CDK1-loaded sEV treatment on changes in the phosphorylation of AKT^{Ser473} and ERK$^{Thr202/Tyr204}$ and observed increased phosphorylation in CDK1-loaded sEV-treated groups consistent with the above immunofluorescent

Figure 2. Testing of the activity of CDK1-loaded sEVs in impaired wound healing

(A) Schematic of CDK1-loaded sEVs used in the single-dose treatment of the wound bed of diabetic obese mice. (B) Representative images of wound bed following topical treatment with PBS, mock sEVs, or CDK1-loaded sEVs. (C) Quantification of wound-closure kinetics (n = 6 per group; *$p < 0.05$; ****$p < 0.0001$). (D) Representative H&E-stained section of wounds collected on day 3 post-treatment with sEVs (top row: low magnification; bottom row: high magnification). (E) Quantification of epithelial thickness based on imaging analysis of H&E-stained sections (n = 6; ****$p < 0.0001$). (F) Localization of Ki67^{+} cells by immunohistochemistry on day 3 post-treatment with sEVs (top row: low magnification; bottom row: high magnification; brown arrows indicate Ki67^{+} staining). (G) Quantification of Ki67^{+} cells shows the number of Ki67^{+} cells per unit area (n = 6; **$p < 0.01$; ***$p < 0.001$,).

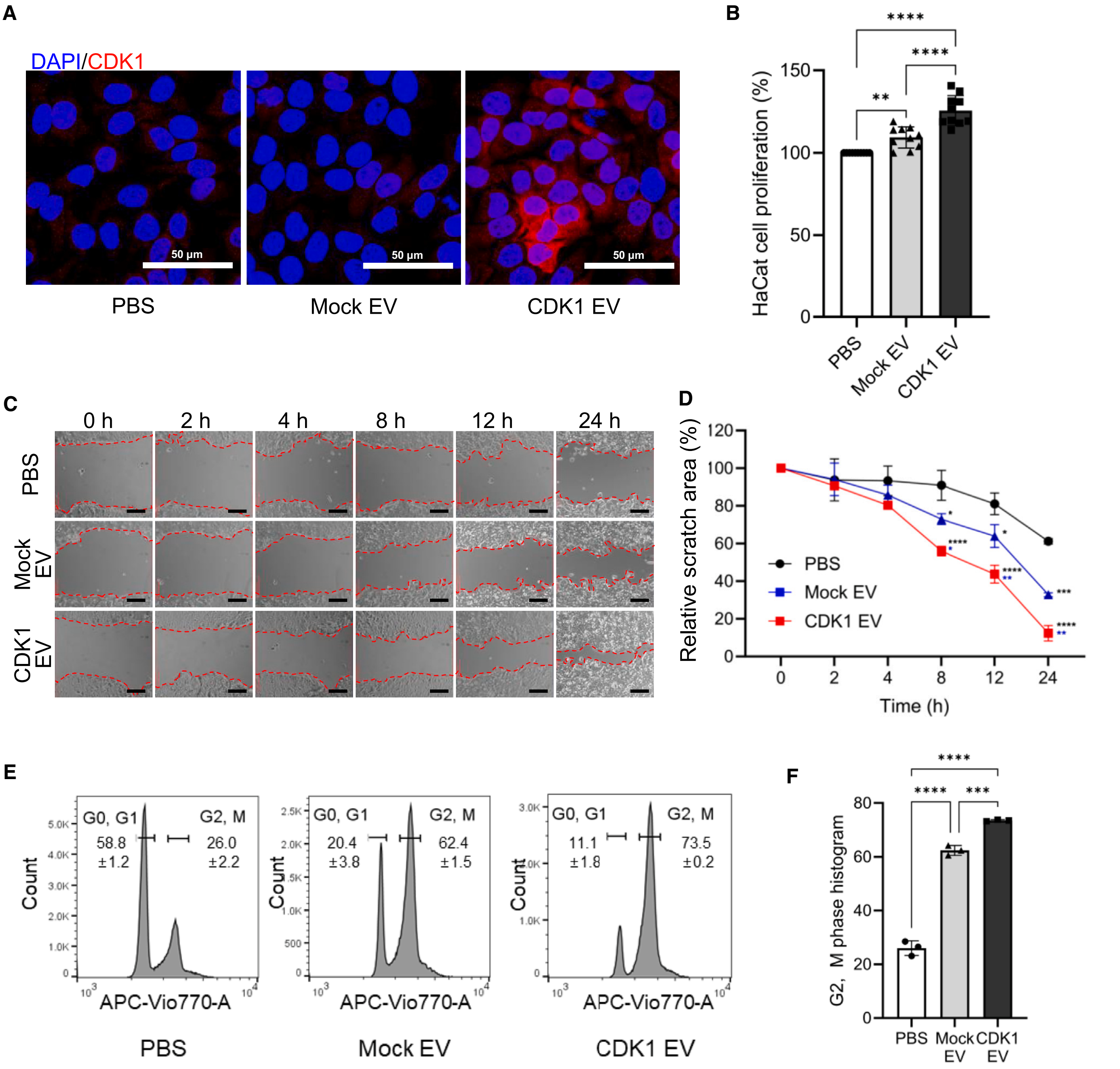

Figure 3. Testing the activity of CDK1-loaded sEVs upon human keratinocytes *in vitro*

(A) Immunofluorescence staining of cells with an anti-CDK1 antibody (red), and counterstained with a nuclear stain (blue) post-sEV treatment (scale bar: 50 μm). (B) Proliferation of human keratinocyte following EV treatment using CCK-8 assay (n = 10; ** $p < 0.01$; **** $p < 0.0001$). (C) Representative imaging of *in vitro* scratch assay in the presence of the proliferation inhibitor mitomycin C following treatment with sEVs and controls (scale bar: 200 μm). (D) Quantification of closure kinetics (n = 4; * $p < 0.05$; ** $p < 0.01$; *** $p < 0.001$; **** $p < 0.0001$). (E) Effect of sEV treatment on cell cycle using a cell-permeable DNA dye and analysis by flow cytometry. (F) Quantification of G2,M phase from sEV-treated cells (n = 3, *** $p < 0.001$; **** $p < 0.0001$).

studies. A similar increase in 4E-BP1$^{Thr37/46}$ phosphorylation was also observed (Figures 4G–4I), supporting a model that CDK1-loaded sEVs stimulate the phosphorylation of several CDK1 pathway molecular endpoints, and led us to consider the potential effects of CDK1 delivery on chromatin structure.

Delivery of CDK1-loaded sEVs promoted histone phosphorylation

To determine whether delivery of CDK1-loaded sEVs affects chromatin remodeling, we focused on monitoring changes in the phosphorylation of histone H3 (pHistone H3^{Ser10}), based on it being a

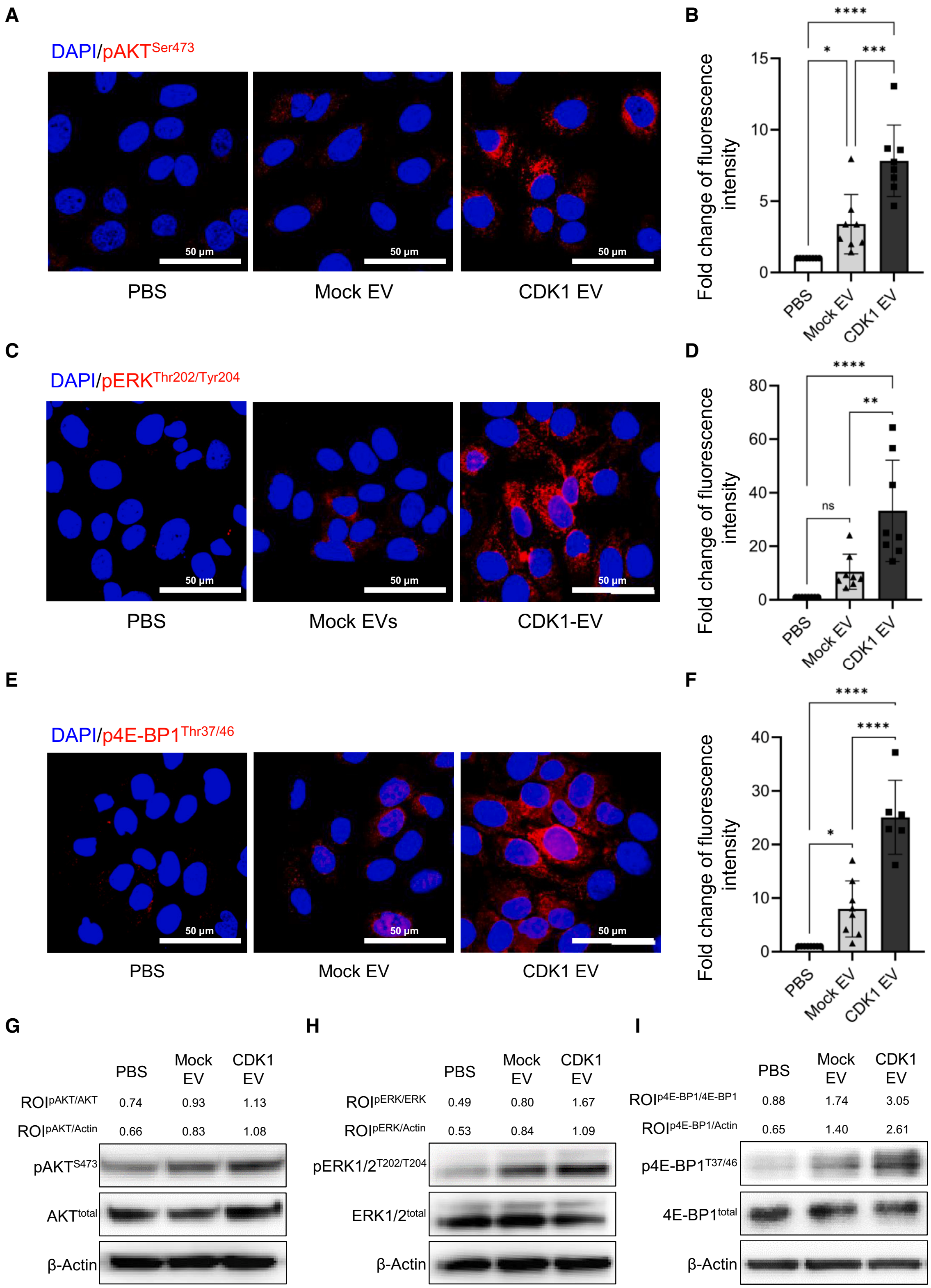

(legend on next page)

target of CDK1 activation through the ERK pathway. Cells were synchronized by serum starvation and sEV-treated cells immunostained with an antibody to pHistone H3^{Ser10}. We observed a 2.8-fold increased pHistone H3^{Ser10} signal in CDK1 EV-treated cells relative to mock EV-treated cells (Figures 5A and 5B). To determine whether delivery of CDK1-loaded sEVs affected progression into specific stages of mitosis, we quantified changes in chromatin structure by enumerating sEV-treated cells in phases of the cell cycle (Figure 5C). We observed that treatment with CDK1-loaded sEVs increased the overall number of cells entering anaphase, whereas PBS-treated and mock sEV-treated cells remained mainly in metaphase and prophase. Our findings show that CDK1-loaded sEVs can deliver biochemically active protein payloads that accelerated wound healing and increased cell proliferation, migration, and cell signaling, which was highlighted by an increased phosphorylation of histone H3 that promoted entry in anaphase (Figure 6).

DISCUSSION

This study utilized sEVs engineered with a CDK1 protein payload to demonstrate the biological and biochemical activity of CDK1 in promoting wound healing and signaling, respectively. We focused on CDK1 because it is highly conserved and because it is the only CDK protein that has been shown to be essential for cell proliferation.[63–66] While CDK1 has an essential role in the regulation of the cell cycle, other CDKs, like CDK6, have been shown to be activated by specific miRNAs to promote wound healing,[67] and delivery of CDK13 in EVs can also promote wound healing.[21] We have previously shown the benefits of actively loading protein payloads into engineered sEVs with techniques such as the myristoylation tag to biochemical validation studies. In the case of CDK1, another likely benefit of this technique is to enhance the stability of delivered proteins and protect it from repression by cyclin binding.[68] Thus, our engineered CDK1-loaded sEVs are able to take advantage of both the protein sorting machinery of EV formation in the multivesicular body and the protection of cargoes to maintain bioactivity.[57] In general, EV cargoes are released into the cytoplasm as a result of endosomal maturation influenced by luminal pH and cholesterol,[56] which we confirmed with our GFP-loaded sEV studies. These studies demonstrate that CDK1-loaded sEVs were internalized into recipient cells and escaped the endosomal pathway to activate effector molecules in the cytosol and nucleus.

There are several interesting questions regarding the biochemical activity of CDK1 in these sEV loading studies. For example, we show that delivery of CDK1-loaded sEVs stimulated the phosphorylation of cytosolic 4E-BP1 and nuclear histone H3. These effectors are downstream of AKT and ERK activation, which are important regulators of proliferation and wound healing.[69] CDK1-mediates increases in 4E-BP1 phosphorylation to regulate global translation downstream, coupling cell proliferation with protein synthesis,[70] which we identified in our study of CDK1-induced proliferation of human keratinocytes. CDK1 is also associated with the regulation of other pathways, such as its interactions with cyclin B and its repression of lysosomal degradation.[71] In this example, CDK1 is also known to inhibit mTOR complex 1 (mTORC1), a regulator of autophagy.[72] It is interesting that CDK1 expression repressed mTORC1 trafficking to lysosomes. In our study, we hypothesize that non-specific mitosis initiated by EV treatment leads to CDK1-dependent arrest of lysosomal degradation that facilitates further EV endosomal escape and the dose-dependent CDK1 activity that we have identified.

Our studies build upon recent reports that focused on loading biologically active pro-reparative protein payloads in sEVs to reverse the impaired wound healing observed in diabetes, infection, and aging. Chronic wounds, particularly in diabetic patients, are highly prevalent and carry significant morbidity and mortality.[3,8] sEV-based therapy has emerged as a promising strategy in promoting the kinetics and durability of wound healing,[73–75] in part because distinct sEVs can promote the various stages of hemostasis, inflammation, proliferation, and remodeling.[76] In this study, a single dose of CDK1-sEVs were administered prior in the inflammatory phase during wound healing to evaluate the effect on cell proliferation of resident cells in the wound area.[77] The potential of sEVs as therapeutics is further supported by low immunogenicity and the ability to harness endogenous sEV release pathways from donor cells and uptake pathways in recipient cells to deliver functional payloads.[78,79] With relatively few studies using engineered EVs to deliver biologically active protein payloads that accelerate wound healing in chronic models,[29,80] our development of the CDK1 strategy that focuses on well-defined molecular endpoints like cell proliferation will likely expand the testing of other relevant pro-reparative EV payloads. CDK1-related factors are thought to be dysregulated in diabetic patients[81] based in part on changes in PI3K/AKT signaling that is impaired in various tissues due to the insulin resistance of diabetic obese patients.[82] In support of this, the CDK1-regulated signaling factor histone H3 is directly phosphorylated at Ser10 by CDK1 via KimH3 phosphorylation or indirectly via the ERK signaling pathway.[44,45] During cell division, histone H3^{Ser10} phosphorylation localizes to the centromeric heterochromatin in late G2 phase, then spreads along the chromosomal arms and throughout the whole chromosome in prophase.[46] In other examples, high glucose, transforming growth factor β, or angiotensin II induce CDK inhibitors such as p21^{Cip1} and p27^{Kip1} that can result in cell-cycle arrest.[83–85] Dysfunction of AKT signaling in diabetic wounds can reduce the phosphorylation of 4E-BP1 and the expression of growth factor.[69,86,87] These examples provide insights into

Figure 4. Downstream signaling mediated by treatment with CDK1-loaded sEVs in human keratinocytes

(A and B) (A) Immunofluorescent localization of p-AKT^{Ser473} and (B) quantification. (C and D) (C) Localization of p-ERK$^{Thr202/Tyr204}$ and (D) quantification. (E and F) (E) Localization of phospho-4E-BP1$^{Thr37/46}$ and (F) quantification (scale bar: 50 μm) ($n = 8$; $^{*}p < 0.05$; $^{***}p < 0.001$; $^{****}p < 0.0001$). (G–I) Immunoblotting for (G) pan-AKT and p-AKT^{Ser473}, (H) pan-ERK and phospho-ERK$^{Thr202/Tyr204}$, and (I) pan-4E-BP1 and phospho-4E-BP1$^{Thr37/46}$ and levels normalized to β-actin.

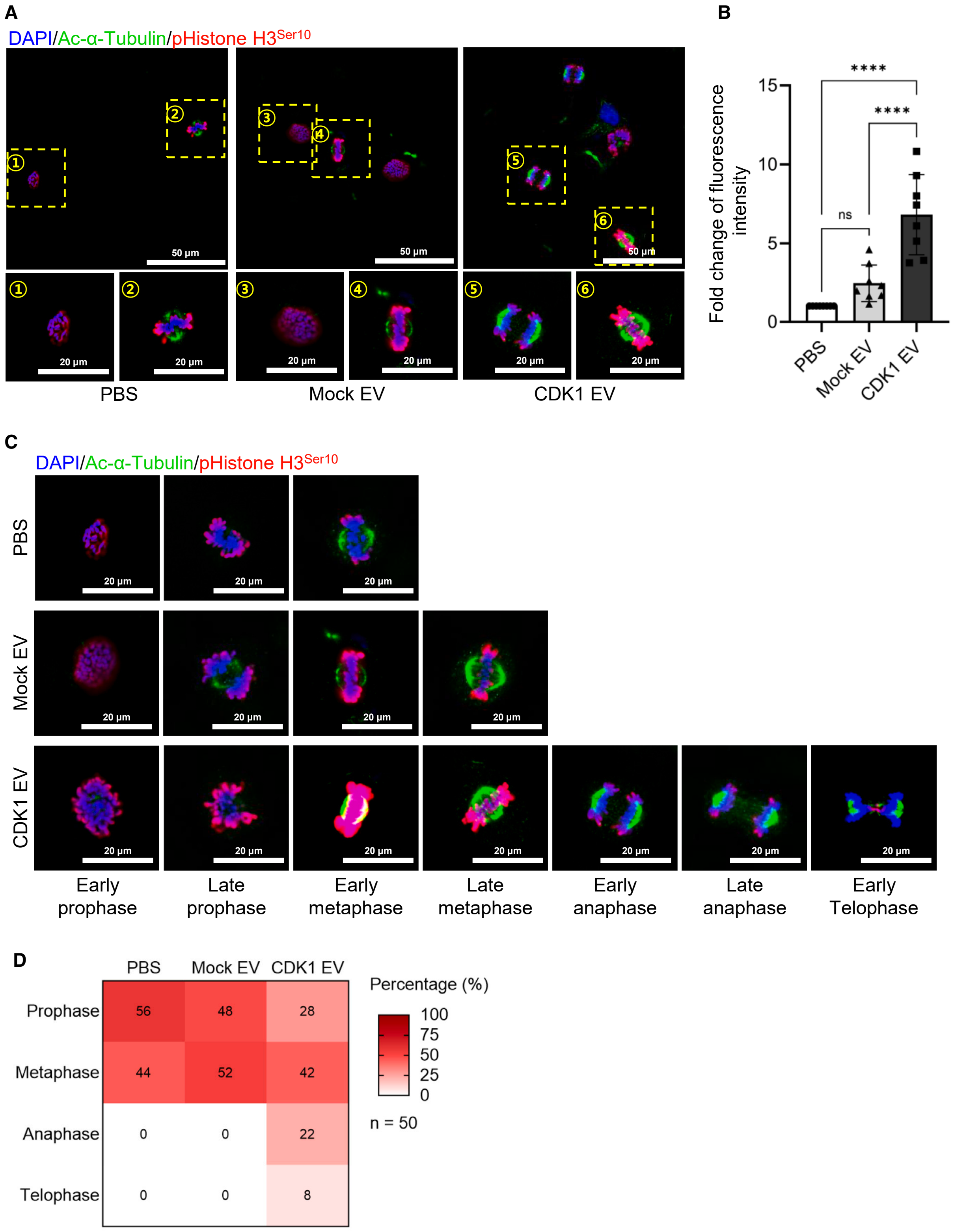

(legend on next page)

the various pathways that CDK1 and its effectors can regulate to restore healthy wound closure.

Downstream substrates of CDK1 may have carcinogenic effects. However, studies on carcinogenesis by exogenous CDK1 in impaired wound healing have not been reported, and the therapeutic effect of CDK1 as a cancer treatment target varies depending on its phosphorylation regulatory site.[53] Studies on this are still unclear. In this study, improved cell proliferation and impaired wound healing by exogenous supplementation of CDK1 delivery by EVs and the effect of CDK1 occurred in a short period of time and were temporary.

Our recent study has shown that pro-reparative EVs stimulate the proliferation of basal keratinocytes, as determined by Ki67 immunohistochemistry.[19] Furthermore, the activity of these engineered sEVs can be demonstrated on isolated human keratinocytes using cell proliferation and signaling readouts. Our studies show that CDK1-loaded sEVs promote keratinocyte migration, proliferation, and signaling, which are critical roles for the keratinocyte in regulating skin homeostasis and inflammation.[88,89] Kinetic studies of wound closure suggest that keratinocytes migrate upon a layer of fibroblasts that provide extracellular matrix to establish a viable proliferative epithelial barrier.[90,91] The effects of CDK1-loaded sEVs upon both cell migration and proliferation suggest that there may be overlapping pathways or that the effects may have different kinetics.[92] CDK1 activity influences not only cell proliferation but also adhesion processes relevant to migration, such as phosphorylation of keratin 5, which are critical for the formation of the basal layer of stratified squamous epithelia.[93,94]

Overall, our study demonstrates that the delivery of a well-defined kinase by sEVs can drive the proliferation and migration of keratinocytes and enhance wound closure. We demonstrate the downstream signaling pathways utilized in this process, expanding our molecular and biochemical understanding of tissue repair, and identify a novel therapeutic strategy for treating non-healing wounds, a great clinical need.

MATERIALS AND METHODS

Cell culture

HEK 293T cells (catalog no. 632180, Takara Bio) were used for transient transfection and EV production. HaCaT cells were used for *in vitro* proliferation assay, cell-cycle assay, and phosphorylation assay. The HEK 293T and HaCaT cells were maintained in Dulbecco's modified Eagle's medium (DMEM; catalog no. 12430054, Gibco) supplemented with 10% (v/v) fetal bovine serum (FBS; catalog no. F0926, Sigma-Aldrich) and 1 × antibiotic-antimycotic (AA; catalog no. 15240062, Gibco) in humid air with 5% CO_2 at 37°C. Cell counting was performed using a hemocytometer with 0.4% trypan blue (catalog no. T10282, Thermo Fisher).

Cloning

Construction of CDK1-EVs was based on the XPack CMV-XP vector (System Biosciences [SBI]) that contained the EV signal peptide as an N-terminal fusion with a multiple cloning site (XPack CMV-XP-MCS, catalog no. XPAK510PA-1, SBI). Primers designed by TAKARA tools (https://www.takarabio.com/learning-centers/cloning/primer-design-and-other-tools) and amplified CDK1 genes from cDNA (catalog no. SC111605, Origene) encoding human CDK1 (NM_001786). PCR product was designed to contain Xho I (catalog no. R0146, New England Biolabs [NEB]) and NotI (catalog no. R0189S, NEB) restriction enzyme sites at the end of the insert gene. The following primers were used for CDK1-F (5′-GCA AAG ATG CCT CGA GGA TGG AAG ATT ATA CCA AAA TAG A-3′) and CDK1-R (5′-AGA ATT CTC GCG GCC GCC TAC ATC TTC TTA ATC TGA TTG T-3′). After transformation into stable competent *Escherichia coli* (catalog no. C3040I, NEB), followed by ampicillin selection, recombinant plasmids were eluted (catalog no. D4203, Zymo Research).

Plasmid transfection

HEK 293T cells were seeded into 100-mm tissue culture-treated dishes, with the numbers of dishes decided by each experiment. Lipofectamine 2000 transfection reagent (catalog no. 11668019, Thermo Fisher) was used for transient transfection of HEK 293T cells to express CDK1 into EVs. The transient transfection was carried out according to the manufacturer's protocol. Briefly, 1 day before transfection, 5×10^6 cells were seeded into each dish with 10 mL culture media. When cell confluence reached 80%, transfection reagent complexes were added. For preparing transfection reagent complexes, 15 μg plasmids (XPack CMV-XP-MCS as mock, XPack CMV-XP-CDK1 and XPack CMV-XP-GFP [catalog no. XPAK530CL-1, SBI]) in the Opi-MEM up to 750 μL (catalog no. 31985062, Gibco) was mixed with 75 μL Lipofectamine reagent in 675 μL Opi-MEM for each dish. Before adding transfection reagent complexes, a plasmids-transfection reagent mixture was incubated at room temperature for complex formation (10 min).

Animal model

All animal experiments were conducted with protocols approved by the Institutional Animal Care and Use Committee of the University of California, San Diego (UCSD). We used 12- to 16-week-old db/db mice (B6.VJS(D)-Lepr^{db}/J mice; JAX# 000697, The Jackson Laboratory) which had a blood glucose level of >300 mg/dL and a body weight >45 g, the criteria for the diabetic obese model. The mice were maintained on a 12-h light/dark cycle.

Figure 5. Analysis of histone phosphorylation by following treatment with CDK1-loaded sEVs onto human keratinocytes

(A) Immunofluorescent imaging to localize p-Histone H3^{Ser10} (red), and counterstained with acetyl-α-tubulin (green) and DAPI for nuclei (blue) following sEV treatment (top row: low magnification; bottom row: high magnification) (scale bars: 50 and 20 μm). (B) Quantification of p-Histone H3^{Ser10} following EV treatment (n = 8; ****p < 0.0001). (C) Representative immunofluorescent images of cell-cycle progression following sEV (scale bar: 20 μm). (D) Distribution of mitotic phases based on each sEV treatment (n = 50 for each treatment).

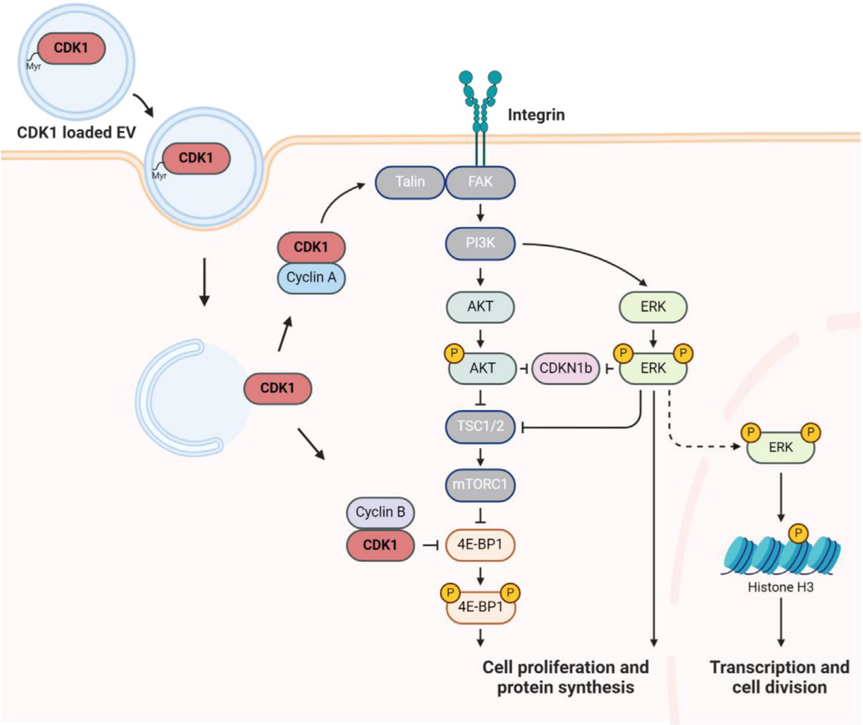

Figure 6. Model for driving entry into the mitotic cycle by treatment of cells with CDK1-loaded sEVs that promote cytosolic signaling and phosphorylation of nuclear histones on chromatin

sEV preparation

EV studies addressed the methodological recommendations of the Minimal Information for Studies of Extracellular Vesicles 2023[51] and reporting that are archived at EV-TRACK. EVs were produced by transient transfection. After 2 days of adding plasmid-transfection reagent complexes, the medium was changed to DMEM supplemented with 10% (v/v) exosome-depleted FBS (catalog no. EXO-FBS-250A-1, SBI) and 1× AA. After 72 h, the conditioned medium (CM) was harvested and centrifuged at 10,000 × g for 10 min, 2 times. The supernatant was filtered through a syringe filter (PES membrane, 0.22 μm; catalog no. 25-244, GenClone). sEVs from the filtered CM were concentrated using Exoquick reagent (catalog no. EXOTC50A-1) following the manufacturer's protocol. The ratio of Exoquick reagent to CM was 1:5 (v/v) in this study. After adding the Exoquick reagent to CM, mixtures were incubated overnight at 4°C and centrifuged at 1,500 × g for 30 min. The supernatant was gently aspirated and residues were centrifuged at 1,500 × g for 5 min and discarded. Precipitated sEVs were resuspended by PBS.

Immunoblotting

After harvesting CM, transfected cells and sEVs were lysis by radioimmunoprecipitation assay lysis buffer (catalog no. 89901, Thermo Fisher) supplemented with 1× protease and phosphatase inhibitor cocktail (Halt protease and phosphatase single-use inhibitor cocktail [100×], catalog no. 78442, Thermo Fisher). The whole-cell lysates (WCLs) and sEVs subjected to immunoblotting were normalized by protein quantification by bicinchoninic acid assay (catalog no. 23225, Thermo Fisher). Samples were prepared in lithium dodecyl sulfate sample buffer (catalog no. NP0008, Thermo Fisher) with 5 mM dithiothreitol (catalog no. 15508013, Thermo Fisher). We loaded 6 μg protein into 12% Bis-Tris Mini Gel (catalog no. NP0342BOX, Thermo Fisher) to separate proteins and transferred it to polyvinylidene fluoride membrane (0.45 μm, catalog no. LC2005, Thermo Fisher). We used 5% non-fat dry milk (catalog no. 9999, Cell Signaling Technology [CST]) in Tris-buffered saline with 0.05% Tween 20 (catalog no. 9997, CST) was used for blocking and primary antibodies (Alix, 1:2,000, catalog no. 92880, CST; Calnexin, 1:2,000, catalog no. 2679, CST; CD63, 1:2,000, catalog no. PA5-92370, Thermo Fisher; CDK1, 1:2,000, catalog no. 9116, CST; CD81, 1:2,000, catalog no. 56039, CST; *p*-CDK1 [Tyr15], 1:2,000, catalog no. 9111, CST; Akt, 1:1,000, catalog no. 4691, CST; *p*-Akt [Ser473], 1:2,000, catalog no. 4060, CST; 4E-BP1, 1:1,000, catalog no. 9644, CST; p-4E-BP1 [Thr37/46], 1:2,000, catalog no. 2855, CST; Erk 1/2, 1:1,000, catalog no. 4695, CST; *p*-Erk 1/2 [Thr202/204], 1:2,000, catalog no. 4370, CST; β-actin, 1:2,000, catalog no. 3700, CST) incubated overnight at 4°C. Secondary antibodies (1:10,000) (anti-rabbit immunoglobulin G [IgG], horseradish peroxidase [HRP]-linked, catalog no. 7074, CST, or anti-mouse IgG, HRP-linked, catalog no. 7076, CST) incubated for 45 min at room temperature with gentle agitation. Immunoblots were detected with HRP-conjugated secondary, incubated with enhanced chemiluminescent reagent (SignalFire Elite ECL reagent, catalog no. 12757, CST), and detected with an IVIS-Lumina Imager (PerkinElmer).

Single vFC

sEV concentration, size distribution, and identification of transmembrane and fluorescent proteins were measured by single vFC using lipophilic fluorescent dye, vFRed (vFC EV Analysis assay kit, catalog no. CBS4HP, Cellarcus Biosciences), using Cytoflex S flow cytometer (Beckman Coulter). The flow cytometer was calibrated for vesicle size using fluorescent intensity standard beads (vCal nanoRainbow Beads, catalog no. CBS6, Cellarcus Biosciences) and antibody capture beads (vCal nanoCal antibody capture beads, Cellarcus Biosciences) to calibrate flow data. Samples were subjected to a 1,000-fold dilution, stained with vFRed and phycoerythrin (PE)-conjugated antibody corresponding to a cocktail of anti-human monoclonal antibodies against human CD9, CD63, and CD81 (vTag anti-human tetraspanin antibody, catalog no. CBS5-PE, Cellarcus Biosciences), and 120 μL measured on the flow cytometer at a flow rate of 60 μL/min for

2 min. Data were analyzed using FCS Express (Dotmatics/De Novo Software) and a standardized layout used to apply gating, compensation, and calibration (Cellarcus Biosciences).

In vivo wound-healing assay in diabetic mice model

For the wound-healing activity of CDK1-loaded sEVs in the db/db mice model, hair was removed by shaving and topical treatment with depilatory cream of dorsal skin, a full-thickness 4-mm punch made (catalog no. P450, Acuderm), and the wound site splinted with a silicone ring (catalog no. GBLRD476687, Grace Bio-Labs) by 4-0 nylon suturing (catalog no. MV-662, Med Vet International). We treated 2.0×10^7 CDK1 and empty (mock) sEVs (in a volume of 50 μL PBS per wound) and covered them with 3M Tegaderm dressing film (catalog no. 1622w). The wound site was imaged with iPhone 12 Pro (ISO 125, 26 mm, 0ev, F1.6, 1/60s) and analyzed by ImageJ (1.54i version, NIH). Tissues were harvested for histology analysis by fixation of skin wound samples in 4% paraformaldehyde into paraffin at the UCSD Tissue Technology Shared Resource (TTSR) that prepared slides stained with hematoxylin and eosin (H&E). Immunohistochemical (IHC) staining to localize Ki67 (1:50, catalog no. 16667, GeneTex) was performed with an Intellipath Automated IHC Stainer (Biocare) by the TTSR. H&E and IHC images were analyzed using Aperio ImageScope version 12.4.6.5003 software (Leica Biosystems).

Immunofluorescence

For detecting CDK1 after CDK1-loaded sEVs treatment, immunofluorescence (IF) was imaged as in the following procedure: 1.0×10^5 HaCaT cells were seeded onto a 12-well plate (black frame 12-well plate with glass-like polymer bottom, catalog no. P12-1.5P, Cellvis) and incubated in a humidified 5% CO_2 incubator at 37°C for 24 h. To synchronize the cell cycle, the cells were starved with culture medium containing 1% FBS and 1× AA for 24 h. We loaded 2.0×10^5–10^8 CDK1-loaded sEVs and 2.0×10^7 empty (mock) sEVs (in a volume of 100 μL PBS per well) onto each well for 6 h. PBS was treated as a negative control. After EV exposure, EV-treated HaCaT cells were washed twice with cold PBS and fixed by 4% paraformaldehyde for 10 min at 37°C. The cells were permeabilized by 0.15% Triton X-100 for 15 min at room temperature. Non-specific antibody binding was blocked by 2.5% BSA for 1 h at room temperature. Primary antibodies (CDK1, 1:400, catalog no. 9116, CST, for *in vitro* study; catalog no. 19532-1-AP, Proteintech, for *in vivo* study; Ki67-FITC, 1:50, catalog no. 130-130-859, Miltenyi Biotec; vimentin, 1:100, catalog no. 5741, CST; Keratin14, 1:200, catalog no. 10143-1-AP, Thermo-Fisher) were incubated overnight at 4°C. Secondary antibodies (4 μg/mL) (anti-mouse IgG, Alexa Fluor 546-linked, catalog no. A11030, Thermo Fisher) were incubated for 45 min at room temperature. Nucleus was stained with 1 μg/mL DAPI for 5 min at room temperature. IFs were imaged with confocal laser scanning microscopy (Model AXR, Nikon). IF images were analyzed by ImageJ version 1.54i software.

Cell proliferation assay

To assess the proliferative effects of CDK1-loaded sEVs on HaCaT cells, a cell proliferation assay was carried out using Cell Counting Kit-8 (CCK-8, catalog no. CK04, Dojindo). We seeded 1×10^3 HaCaT cells into a 96-well plate and incubated in a humidified 5% CO_2 incubator at 37°C for 24 h. We treated 2.0×10^6 CDK1 and empty (mock) sEVs in each well for 24 h. The cells treated with PBS were used as a control group. After 24 h, a CCK-8 solution containing the water-soluble tetrazolium salt was added to each well, and the plate was incubated for 2 h. Absorbance was measured using a microplate reader at 450 nm. Relative proliferation was calculated as a percentage to the PBS control group.

Migration assay

We seeded 5×10^3 HaCaT cells using a 2-well silicone insert (catalog no. 80209, Ibidi) in a 24-well plate and incubated in a humidified 5% CO_2 incubator at 37°C for 24 h. To inhibit cell proliferation, 10 μg/mL mitomycin C (catalog no. M0440, Sigma-Aldrich) was treated to HaCaT cells for 2 h and the media replaced with sEVs (2.0×10^7 CDK1- and empty [mock])-containing media for 24 h. The gap between 2-well was imaged over 24 h using a CCD camera (Retiga R6, Teledyne Photometrics) on a microscope (IX70, Olympus) to measure cell migration. All images were analyzed using ZEN blue version 3.4.91.00000 (Carl Zeiss Microscopy GmbH).

Cell-cycle assay

To determine the activity of CDK1-loaded sEVs in the cell cycle, a cell-cycle assay was carried out using membrane-permeable DNA staining solution (cell-cycle assay solution deep red, catalog no. C548, Dojindo). We seeded 3.0×10^5 HaCaT cells into a 6-well plate and incubated the cells in a humidified 5% CO_2 incubator at 37°C for 24 h. To synchronize the cell cycle, the cells were starved with culture medium containing 1% FBS and 1× AA for 24 h. We treated into each well 2.0×10^7 CDK1 and empty (mock) sEVs (in a volume of 100 μL PBS per well) for 3 h with fresh culture medium containing 10% FBS and 1× AA. PBS was treated as a negative control. After EV exposure, EV-treated HaCaT cells were washed with cold PBS two times and suspended. Cell suspension was washed by PBS twice and resuspended in 500 μL PBS containing 5 μL cell-cycle assay solution deep red. DNA staining was carried out for 15 min at 37°C and protected from light. The stained cells were analyzed by FC (MACSQuant Analyzer 10, Miltenyi-Biotec). The data were analyzed by FlowJo software version 10.8.2 (BD Biosciences).

In vitro tracking EV uptake

Intracellular EV tracking was carried out by GFP-loaded sEVs. We treated 2.0×10^7 GFP-loaded sEVs (in a volume of 100 μL PBS per well) to 1.0×10^5 HaCaT cells for 3, 6, 12, and 24 h. After EVs exposure, EVs were discarded and washed by cold PBS twice. To determine a co-localization of GFP-tagged EVs with lysosomes, lysosomes were stained with LysoTracker Red DND-99 (catalog no. L7528, Thermo Fisher) for 30 min at 37°C. The cells were fixed by 4% paraformaldehyde for 10 min at 37°C. IFs were imaged with confocal laser scanning microscopy (Model AXR, Nikon). IF images were analyzed by ImageJ version 1.54i software.

Signaling pathway assay

We seeded 1.0×10^5 HaCaT cells into 12-well plate (black frame 12-well plate with glass-like polymer bottom, catalog no. P12-1.5P, Cellvis) and incubated them in a humidified 5% CO_2 incubator at 37°C for 24 h. To synchronize the cell cycle, the cells were starved with culture medium containing 1% FBS and 1× AA for 24 h. We treated into each cell 2.0×10^7 CDK1 and empty (mock) sEVs (in a volume of 100 μL PBS per well) for 1 h (immunoblotting) and 6 h (IF). PBS was treated as a negative control. After EV exposure, the phosphorylations of Akt, 4E-BP1, and Erk were assessed by immunoblotting and IF.

For IF, sEV-treated HaCaT cells were washed with cold PBS twice and fixed by 4% paraformaldehyde for 10 min at 37°C. The cells were permeabilized by 0.15% Triton X-100 for 15 min at room temperature. Non-specific antibody binding was blocked by 2.5% BSA for 1 h at room temperature. Primary antibodies (p-Akt [Ser473], 1:400, catalog no. 4060, CST; p-4E-BP1 [Thr37/46], 1:400, catalog no. 2855, CST; p-Erk 1/2 [Thr202/204], 1:400, catalog no. 4370, CST; $p27^{Kip1}$, 1:800, catalog no. 3686, CST; pHistone H3 [Ser10], 1:200, catalog no. 9706, CST; acetyl-α-tubulin, 1:800, catalog no. 5335, CST) were incubated overnight at 4°C. Secondary antibodies (4 μg/mL) (anti-rabbit IgG, Alexa Fluor 488 linked, catalog no. A11008, Thermo Fisher; anti-rabbit IgG, Alexa Fluor 546 linked, catalog no. A11010, Thermo Fisher; anti-mouse IgG, Alexa Fluor 488 linked, catalog no. A11029, Thermo Fisher; anti-mouse IgG, Alexa Fluor 546 linked, catalog no. A11030, Thermo Fisher) were incubated for 45 min at room temperature. Nucleus was stained by 1 μg/mL DAPI for 5 min at room temperature. IFs were imaged with confocal laser scanning microscopy (Model AXR, Nikon). IF images were analyzed by ImageJ version 1.54i.

Statistical analysis

All statistical analyses were performed with GraphPad Prism 10.0 (GraphPad Software). Data were expressed as the mean (standard deviation [SD]). Differences between different groups were compared by one-way ANOVA and two-way ANOVA with multiple comparisons with statistically significant p values indicated as $^{*}p < 0.05$; $^{**}p < 0.005$; $^{***}p < 0.001$; $^{****}p < 0.0001$. All statistical analyses and representative images presented and observed in at least three independent experiments.

DATA AND CODE AVAILABILITY

All reagents and supporting data, including instrument controls and other reasonable requests are available from the corresponding author.

ACKNOWLEDGMENTS

Katie Pool provided expert technical support. The UCSD Biorepository and Tissue Technology Shared Resources (BTTSR) core at the Moores Cancer Center provided histology services. Confocal microscopy was performed at the Nikon Imaging Center at UCSD. Schematics and the graphical abstract were created in Biorender.com under license agreement. This work was supported by National Institutes of Health grants (1R01GM140137 and 1R35GM149245, to B.P.E.) and KL2TR001444 of the NIH Clinical and Translational Science Award funding through the UCSD Clinical Translational Research Institute (to R.A.D.).

AUTHOR CONTRIBUTIONS

Conceptualization, W.C. and B.P.E.; methodology, W.C., D.J.P., R.A.D., K.N., and M.Y.; software, W.C.; validation, W.C.; investigation, W.C., D.J.P., R.A.D., K.N., M.Y., and B.P.E.; resources, W.C.; data curation, W.C.; writing – original draft, W.C. and B.P.E.; visualization, W.C. and B.P.E.; funding acquisition, R.A.D. and B.P.E.

DECLARATION OF INTERESTS

The laboratory receives funding support from Ionis Pharmaceuticals (to B.P.E.) for EV tropism screening research that is unrelated to the studies described here. B.P.E. is a co-founder of Saragen Therapeutics.

SUPPLEMENTAL INFORMATION

Supplemental information can be found online at https://doi.org/10.1016/j.ymthe.2025.01.039.

REFERENCES

1. Guan, Y., Niu, H., Liu, Z., Dang, Y., Shen, J., Zayed, M., Ma, L., and Guan, J. (2021). Sustained oxygenation accelerates diabetic wound healing by promoting epithelialization and angiogenesis and decreasing inflammation. Sci. Adv. *7*, eabj0153.
2. McDermott, K., Fang, M., Boulton, A.J.M., Selvin, E., and Hicks, C.W. (2023). Etiology, Epidemiology, and Disparities in the Burden of Diabetic Foot Ulcers. Diabetes Care *46*, 209–221. https://doi.org/10.2337/dci22-0043.
3. Dasari, N., Jiang, A., Skochdopole, A., Chung, J., Reece, E.M., Vorstenbosch, J., and Winocour, S. (2021). Updates in Diabetic Wound Healing, Inflammation, and Scarring. Semin. Plast. Surg. *35*, 153–158. https://doi.org/10.1055/s-0041-1731460.
4. Koh, T.J., and DiPietro, L.A. (2011). Inflammation and wound healing: the role of the macrophage. Expert Rev. Mol. Med. *13*, e23. https://doi.org/10.1017/S1462399411001943.
5. Gao, M., Nguyen, T.T., Suckow, M.A., Wolter, W.R., Gooyit, M., Mobashery, S., and Chang, M. (2015). Acceleration of diabetic wound healing using a novel protease-anti-protease combination therapy. Proc. Natl. Acad. Sci. USA *112*, 15226–15231. https://doi.org/10.1073/pnas.1517847112.
6. Pang, J., Maienschein-Cline, M., and Koh, T.J. (2021). Enhanced Proliferation of Ly6C(+) Monocytes/Macrophages Contributes to Chronic Inflammation in Skin Wounds of Diabetic Mice. J. Immunol. *206*, 621–630. https://doi.org/10.4049/jimmunol.2000935.
7. Khunger, N. (2017). Accelerated Wound Healing: Harnessing the Power of Platelets, Biomaterials, Stem Cells and Gene Therapy (Medknow).
8. Burgess, J.L., Wyant, W.A., Abdo Abujamra, B., Kirsner, R.S., and Jozic, I. (2021). Diabetic Wound-Healing Science. Medicina (Kaunas) *57*, 1072. https://doi.org/10.3390/medicina57101072.
9. Michowski, W., Chick, J.M., Chu, C., Kolodziejczyk, A., Wang, Y., Suski, J.M., Abraham, B., Anders, L., Day, D., Dunkl, L.M., et al. (2020). Cdk1 Controls Global Epigenetic Landscape in Embryonic Stem Cells. Mol. Cell *78*, 459–476.e13. https://doi.org/10.1016/j.molcel.2020.03.010.
10. Diril, M.K., Ratnacaram, C.K., Padmakumar, V.C., Du, T., Wasser, M., Coppola, V., Tessarollo, L., and Kaldis, P. (2012). Cyclin-dependent kinase 1 (Cdk1) is essential for cell division and suppression of DNA re-replication but not for liver regeneration. Proc. Natl. Acad. Sci. USA *109*, 3826–3831. https://doi.org/10.1073/pnas.1115201109.
11. Ferroni, L., Gardin, C., Dalla Paola, L., Campo, G., Cimaglia, P., Bellin, G., Pinton, P., and Zavan, B. (2019). Characterization of Dermal Stem Cells of Diabetic Patients. Cells *8*, 729. https://doi.org/10.3390/cells8070729.
12. Ow, J.R., Caldez, M.J., Zafer, G., Foo, J.C., Li, H.Y., Ghosh, S., Wollmann, H., Cazenave-Gassiot, A., Ong, C.B., Wenk, M.R., et al. (2020). Remodeling of whole-body lipid metabolism and a diabetic-like phenotype caused by loss of CDK1 and hepatocyte division. Elife *9*, e63835. https://doi.org/10.7554/eLife.63835.
13. Bare, Y., Chan, G.K., Hayday, T., McGrath, J.A., and Parsons, M. (2021). Slac2-b Coordinates Extracellular Vesicle Secretion to Regulate Keratinocyte Adhesion and Migration. J. Invest. Dermatol. *141*, 523–532.e2. https://doi.org/10.1016/j.jid.2020.08.011.

14. Flemming, J.P., Wermuth, P.J., and Mahoney, M.G. (2024). Extracellular Vesicles in the Skin Microenvironment: Emerging Roles as Biomarkers and Therapeutic Tools in Dermatologic Health and Disease. J. Invest. Dermatol. *144*, 225–233. https://doi.org/10.1016/j.jid.2023.08.024.

15. Wiklander, O.P.B., Brennan, M.Á., Lötvall, J., Breakefield, X.O., and El Andaloussi, S. (2019). Advances in therapeutic applications of extracellular vesicles. Sci. Transl. Med. *11*, eaav8521.

16. Hettich, B.F., Ben-Yehuda Greenwald, M., Werner, S., and Leroux, J.C. (2020). Exosomes for Wound Healing: Purification Optimization and Identification of Bioactive Components. Adv. Sci. *7*, 2002596. https://doi.org/10.1002/advs.202002596.

17. Kim, J., Kim, E.H., Lee, H., Sung, J.H., and Bang, O.Y. (2023). Clinical-Scale Mesenchymal Stem Cell-Derived Extracellular Vesicle Therapy for Wound Healing. Int. J. Mol. Sci. *24*, 4273. https://doi.org/10.3390/ijms24054273.

18. Yang, H., Xu, H., Wang, Z., Li, X., Wang, P., Cao, X., Xu, Z., Lv, D., Rong, Y., Chen, M., et al. (2023). Analysis of miR-203a-3p/SOCS3-mediated induction of M2 macrophage polarization to promote diabetic wound healing based on epidermal stem cell-derived exosomes. Diabetes Res. Clin. Pract. *197*, 110573. https://doi.org/10.1016/j.diabres.2023.110573.

19. Park, D.J., Choi, W., Sayeed, S., Dorschner, R.A., Rainaldi, J., Ho, K., Kezios, J., Nolan, J.P., Mali, P., and Costantini, T. (2024). Defining the activity of pro-reparative extracellular vesicles in wound healing based on miRNA payloads and cell type-specific lineage mapping. Mol. Ther. *32*, 3059–3079.

20. Zhang, L., Ouyang, P., He, G., Wang, X., Song, D., Yang, Y., and He, X. (2021). Exosomes from microRNA-126 overexpressing mesenchymal stem cells promote angiogenesis by targeting the PIK3R2-mediated PI3K/Akt signalling pathway. J. Cell. Mol. Med. *25*, 2148–2162. https://doi.org/10.1111/jcmm.16192.

21. Huang, Q., Chu, Z., Wang, Z., Li, Q., Meng, S., Lu, Y., Ma, K., Cui, S., Hu, W., Zhang, W., et al. (2024). circCDK13-loaded small extracellular vesicles accelerate healing in preclinical diabetic wound models. Nat. Commun. *15*, 3904. https://doi.org/10.1038/s41467-024-48284-3.

22. Wang, P., Theocharidis, G., Vlachos, I.S., Kounas, K., Lobao, A., Shu, B., Wu, B., Xie, J., Hu, Z., Qi, S., et al. (2022). Exosomes Derived from Epidermal Stem Cells Improve Diabetic Wound Healing. J. Invest. Dermatol. *142*, 2508–2517.e13. https://doi.org/10.1016/j.jid.2022.01.030.

23. Chevillet, J.R., Kang, Q., Ruf, I.K., Briggs, H.A., Vojtech, L.N., Hughes, S.M., Cheng, H.H., Arroyo, J.D., Meredith, E.K., Gallichotte, E.N., et al. (2014). Quantitative and stoichiometric analysis of the microRNA content of exosomes. Proc. Natl. Acad. Sci. USA *111*, 14888–14893.

24. Toh, W.S., Lai, R.C., Zhang, B., and Lim, S.K. (2018). MSC exosome works through a protein-based mechanism of action. Biochem. Soc. Trans. *46*, 843–853.

25. Roefs, M.T., Sluijter, J.P.G., and Vader, P. (2020). Extracellular vesicle-associated proteins in tissue repair. Trends Cell Biol. *30*, 990–1013.

26. Tang, T.-T., Wang, B., Wu, M., Li, Z.-L., Feng, Y., Cao, J.-Y., Yin, D., Liu, H., Tang, R.-N., Crowley, S.D., et al. (2020). Extracellular vesicle–encapsulated IL-10 as novel nanotherapeutics against ischemic AKI. Sci. Adv. *6*, eaaz0748.

27. Sheller-Miller, S., Radnaa, E., Yoo, J.-K., Kim, E., Choi, K., Kim, Y., Kim, Y.N., Richardson, L., Choi, C., and Menon, R. (2021). Exosomal delivery of NF-κB inhibitor delays LPS-induced preterm birth and modulates fetal immune cell profile in mouse models. Sci. Adv. *7*, eabd3865.

28. Haney, M.J., Klyachko, N.L., Zhao, Y., Gupta, R., Plotnikova, E.G., He, Z., Patel, T., Piroyan, A., Sokolsky, M., Kabanov, A.V., and Batrakova, E.V. (2015). Exosomes as drug delivery vehicles for Parkinson's disease therapy. J. Control Release *207*, 18–30.

29. Park, D.J., Duggan, E., Ho, K., Dorschner, R.A., Dobke, M., Nolan, J.P., and Eliceiri, B.P. (2022). Serpin-loaded extracellular vesicles promote tissue repair in a mouse model of impaired wound healing. J. Nanobiotechnology *20*, 474. https://doi.org/10.1186/s12951-022-01656-7.

30. Zhu, X., Badawi, M., Pomeroy, S., Sutaria, D.S., Xie, Z., Baek, A., Jiang, J., Elgamal, O.A., Mo, X., Perle, K.L., et al. (2017). Comprehensive toxicity and immunogenicity studies reveal minimal effects in mice following sustained dosing of extracellular vesicles derived from HEK293T cells. J. Extracell. Vesicles *6*, 1324730. https://doi.org/10.1080/20013078.2017.1324730.

31. Cheng, K., and Kalluri, R. (2023). Guidelines for clinical translation and commercialization of extracellular vesicles and exosomes based therapeutics. Extracellular Vesicle *2*, 100029.

32. Bloom, J., and Cross, F.R. (2007). Multiple levels of cyclin specificity in cell-cycle control. Nat. Rev. Mol. Cell Biol. *8*, 149–160.

33. Wang, X.Q., Lo, C.M., Chen, L., Ngan, E.S.W., Xu, A., and Poon, R.Y. (2017). CDK1-PDK1-PI3K/Akt signaling pathway regulates embryonic and induced pluripotency. Cell Death Differ. *24*, 38–48. https://doi.org/10.1038/cdd.2016.84.

34. Kamranvar, S.A., Rani, B., and Johansson, S. (2022). Cell Cycle Regulation by Integrin-Mediated Adhesion. Cells *11*, 2521. https://doi.org/10.3390/cells11162521.

35. Chambard, J.C., Lefloch, R., Pouysségur, J., and Lenormand, P. (2007). ERK implication in cell cycle regulation. Biochim. Biophys. Acta *1773*, 1299–1310. https://doi.org/10.1016/j.bbamcr.2006.11.010.

36. Schirripa, A., Sexl, V., and Kollmann, K. (2022). Cyclin-dependent kinase inhibitors in malignant hematopoiesis. Front. Oncol. *12*, 916682. https://doi.org/10.3389/fonc.2022.916682.

37. Gingras, A.-C., Gygi, S.P., Raught, B., Polakiewicz, R.D., Abraham, R.T., Hoekstra, M.F., Aebersold, R., and Sonenberg, N. (1999). Regulation of 4E-BP1 phosphorylation: a novel two-step mechanism. Genes Dev. *13*, 1422–1437.

38. Qin, X., Jiang, B., and Zhang, Y. (2016). 4E-BP1, a multifactor regulated multifunctional protein. Cell Cycle *15*, 781–786. https://doi.org/10.1080/15384101.2016.1151581.

39. Shah, O.J., Kimball, S.R., and Jefferson, L.S. (2000). Acute attenuation of translation initiation and protein synthesis by glucocorticoids in skeletal muscle. Am. J. Physiol. Endocrinol. Metab. *278*, E76–E82.

40. Kleijn, M., and Proud, C.G. (2000). The activation of eukaryotic initiation factor (eIF) 2B by growth factors in PC12 cells requires MEK/ERK signalling. FEBS Lett. *476*, 262–265. https://doi.org/10.1016/s0014-5793(00)01743-9.

41. Shuda, M., Velásquez, C., Cheng, E., Cordek, D.G., Kwun, H.J., Chang, Y., and Moore, P.S. (2015). CDK1 substitutes for mTOR kinase to activate mitotic cap-dependent protein translation. Proc. Natl. Acad. Sci. USA *112*, 5875–5882. https://doi.org/10.1073/pnas.1505787112.

42. Shuda, M., Chang, Y., and Moore, P.S. (2015). Mitotic 4E-BP1 hyperphosphorylation and cap-dependent translation. Cell Cycle *14*, 3005–3006. https://doi.org/10.1080/15384101.2015.1084192.

43. Velasquez, C., Cheng, E., Shuda, M., Lee-Oesterreich, P.J., Pogge von Strandmann, L., Gritsenko, M.A., Jacobs, J.M., Moore, P.S., and Chang, Y. (2016). Mitotic protein kinase CDK1 phosphorylation of mRNA translation regulator 4E-BP1 Ser83 may contribute to cell transformation. Proc. Natl. Acad. Sci. USA *113*, 8466–8471. https://doi.org/10.1073/pnas.1607768113.

44. Crosio, C., Fimia, G.M., Loury, R., Kimura, M., Okano, Y., Zhou, H., Sen, S., Allis, C.D., and Sassone-Corsi, P. (2002). Mitotic phosphorylation of histone H3: spatio-temporal regulation by mammalian Aurora kinases. Mol. Cell. Biol. *22*, 874–885.

45. Wang, J., Tian, X., Feng, C., Song, C., Yu, B., Wang, Y., Ji, X., and Zhang, X. (2023). Histone H3 phospho-regulation by KimH3 in both interphase and mitosis. iScience *26*, 106372. https://doi.org/10.1016/j.isci.2023.106372.

46. Hendzel, M.J., Wei, Y., Mancini, M.A., Van Hooser, A., Ranalli, T., Brinkley, B.R., Bazett-Jones, D.P., and Allis, C.D. (1997). Mitosis-specific phosphorylation of histone H3 initiates primarily within pericentromeric heterochromatin during G2 and spreads in an ordered fashion coincident with mitotic chromosome condensation. Chromosoma *106*, 348–360.

47. Li, Z., Liu, J., Song, J., Yin, Z., Zhou, F., Shen, H., Wang, G., and Su, J. (2024). Multifunctional hydrogel-based engineered extracellular vesicles delivery for complicated wound healing. Theranostics *14*, 4198–4217.

48. Liu, H., Song, P., Zhang, H., Zhou, F., Ji, N., Wang, M., Zhou, G., Han, R., Liu, X., Weng, W., et al. (2024). Synthetic biology-based bacterial extracellular vesicles displaying BMP-2 and CXCR4 to ameliorate osteoporosis. J. Extracell. Vesicles *13*, e12429.

49. Shen, B., Wu, N., Yang, J.-M., and Gould, S.J. (2011). Protein targeting to exosomes/microvesicles by plasma membrane anchors. J. Biol. Chem. *286*, 14383–14395.

50. Yang, J.-M., and Gould, S.J. (2013). The cis-acting signals that target proteins to exosomes and microvesicles. Biochem. Soc. Trans. *41*, 277–282.

51. Welsh, J.A., Goberdhan, D.C.I., O'Driscoll, L., Buzas, E.I., Blenkiron, C., Bussolati, B., Cai, H., Di Vizio, D., Driedonks, T.A.P., Erdbrügger, U., et al. (2024). Minimal information for studies of extracellular vesicles (MISEV2023): From basic to advanced approaches. J. Extracell. Vesicles *13*, e12404. https://doi.org/10.1002/jev2.12404.

52. Welsh, J.A., Van Der Pol, E., Arkesteijn, G.J.A., Bremer, M., Brisson, A., Coumans, F., Dignat-George, F., Duggan, E., Ghiran, I., Giebel, B., et al. (2020). MIFlowCyt-EV: A framework for standardized reporting of extracellular vesicle flow cytometry experiments. J. Extracell. Vesicles *9*, 1713526.

53. Massacci, G., Perfetto, L., and Sacco, F. (2023). The Cyclin-dependent kinase 1: more than a cell cycle regulator. Br. J. Cancer *129*, 1707–1716. https://doi.org/10.1038/s41416-023-02468-8.

54. Dhall, S., Do, D.C., Garcia, M., Kim, J., Mirebrahim, S.H., Lyubovitsky, J., Lonardi, S., Nothnagel, E.A., Schiller, N., and Martins-Green, M. (2014). Generating and reversing chronic wounds in diabetic mice by manipulating wound redox parameters. J. Diabetes Res. *2014*, 562625.

55. Nascimento-Filho, C.H.V., Silveira, E.J.D., Goloni-Bertollo, E.M., de Souza, L.B., Squarize, C.H., and Castilho, R.M. (2020). Skin wound healing triggers epigenetic modifications of histone H4. J. Transl. Med. *18*, 138.

56. Joshi, B.S., de Beer, M.A., Giepmans, B.N.G., and Zuhorn, I.S. (2020). Endocytosis of Extracellular Vesicles and Release of Their Cargo from Endosomes. ACS Nano *14*, 4444–4455. https://doi.org/10.1021/acsnano.9b10033.

57. Choi, W., Park, D.J., and Eliceiri, B.P. (2024). Defining tropism and activity of natural and engineered extracellular vesicles. Front. Immunol. *15*, 1363185.

58. Herbert, T.P., Tee, A.R., and Proud, C.G. (2002). The extracellular signal-regulated kinase pathway regulates the phosphorylation of 4E-BP1 at multiple sites. J. Biol. Chem. *277*, 11591–11596.

59. Manning, B.D., and Toker, A. (2017). AKT/PKB signaling: navigating the network. Cell *169*, 381–405.

60. Fujita, N., Sato, S., Katayama, K., and Tsuruo, T. (2002). Akt-dependent phosphorylation of p27Kip1promotes binding to 14-3-3 and cytoplasmic localization. J. Biol. Chem. *277*, 28706–28713.

61. Villanueva, J., Yung, Y., Walker, J.L., and Assoian, R.K. (2007). ERK activity and G1 phase progression: identifying dispensable versus essential activities and primary versus secondary targets. Mol. Biol. Cell *18*, 1457–1463.

62. Kress, T.R., Raabe, T., and Feller, S.M. (2010). High Erk activity suppresses expression of the cell cycle inhibitor p27Kip1 in colorectal cancer cells. Cell Commun. Signal. *8*, 1–7.

63. Santamaría, D., Barrière, C., Cerqueira, A., Hunt, S., Tardy, C., Newton, K., Cáceres, J.F., Dubus, P., Malumbres, M., and Barbacid, M. (2007). Cdk1 is sufficient to drive the mammalian cell cycle. Nature *448*, 811–815.

64. Malumbres, M., and Barbacid, M. (2009). Cell cycle, CDKs and cancer: a changing paradigm. Nat. Rev. Cancer *9*, 153–166.

65. Saito, M., Mulati, M., Talib, S.Z.A., Kaldis, P., Takeda, S., Okawa, A., and Inose, H. (2016). The Indispensable Role of Cyclin-Dependent Kinase 1 in Skeletal Development. Sci. Rep. *6*, 20622. https://doi.org/10.1038/srep20622.

66. Kalous, J., Jansova, D., and Susor, A. (2020). Role of Cyclin-Dependent Kinase 1 in Translational Regulation in the M-Phase. Cells *9*, 1568. https://doi.org/10.3390/cells9071568.

67. Liao, X., Yan, F., Hu, S., Mu, J., Li, S., He, Y., Tang, M., Chen, J., Yu, L., and Sun, J. (2022). Adipose mesenchymal stem cell sheets-derived extracellular vesicles-microRNA-10b promote skin wound healing by elevating expression of CDK6. Biomater. Adv. *136*, 212781. https://doi.org/10.1016/j.bioadv.2022.212781.

68. Brown, N.R., Korolchuk, S., Martin, M.P., Stanley, W.A., Moukhametzianov, R., Noble, M.E.M., and Endicott, J.A. (2015). CDK1 structures reveal conserved and unique features of the essential cell cycle CDK. Nat. Commun. *6*, 6769. https://doi.org/10.1038/ncomms7769.

69. Huang, H., Cui, W., Qiu, W., Zhu, M., Zhao, R., Zeng, D., Dong, C., Wang, X., Guo, W., Xing, W., et al. (2015). Impaired wound healing results from the dysfunction of the Akt/mTOR pathway in diabetic rats. J. Dermatol. Sci. *79*, 241–251. https://doi.org/10.1016/j.jdermsci.2015.06.002.

70. Haneke, K., Schott, J., Lindner, D., Hollensen, A.K., Damgaard, C.K., Mongis, C., Knop, M., Palm, W., Ruggieri, A., and Stoecklin, G. (2020). CDK1 couples proliferation with protein synthesis. J. Cell Biol. *219*, e201906147. https://doi.org/10.1083/jcb.201906147.

71. Cota, C.D., Dreier, M.S., Colgan, W., Cha, A., Sia, T., and Davidson, B. (2021). Cyclin-dependent Kinase 1 and Aurora Kinase choreograph mitotic storage and redistribution of a growth factor receptor. PLoS Biol. *19*, e3001029. https://doi.org/10.1371/journal.pbio.3001029.

72. Odle, R.I., Florey, O., Ktistakis, N.T., and Cook, S.J. (2021). CDK1, the Other 'Master Regulator' of Autophagy. Trends Cell Biol. *31*, 95–107. https://doi.org/10.1016/j.tcb.2020.11.001.

73. Oh, E.J., Gangadaran, P., Rajendran, R.L., Kim, H.M., Oh, J.M., Choi, K.Y., Chung, H.Y., and Ahn, B.C. (2021). Extracellular vesicles derived from fibroblasts promote wound healing by optimizing fibroblast and endothelial cellular functions. Stem Cells *39*, 266–279. https://doi.org/10.1002/stem.3310.

74. Jiang, M., Jiang, X., Li, H., Zhang, C., Zhang, Z., Wu, C., Zhang, J., Hu, J., and Zhang, J. (2023). The role of mesenchymal stem cell-derived EVs in diabetic wound healing. Front. Immunol. *14*, 1136098. https://doi.org/10.3389/fimmu.2023.1136098.

75. Garima, Sharma, D., Kumar, A., and Mostafavi, E. (2023). Extracellular vesicle-based biovectors in chronic wound healing: Biogenesis and delivery approaches. Mol. Ther. Nucleic Acids *32*, 822–840. https://doi.org/10.1016/j.omtn.2023.05.002.

76. Narauskaite, D., Vydmantaite, G., Rusteikaite, J., Sampath, R., Rudaityte, A., Stasyte, G., Aparicio Calvente, M.I., and Jekabsone, A. (2021). Extracellular Vesicles in Skin Wound Healing. Pharmaceuticals (Basel) *14*, 811. https://doi.org/10.3390/ph14080811.

77. Akhtari, N., Ahmadi, M., Kiani Doust Vaghe, Y., Asadian, E., Behzad, S., Vatanpour, H., and Ghorbani-Bidkorpeh, F. (2024). Natural agents as wound-healing promoters. Inflammopharmacology *32*, 101–125.

78. Herrmann, I.K., Wood, M.J.A., and Fuhrmann, G. (2021). Extracellular vesicles as a next-generation drug delivery platform. Nat. Nanotechnol. *16*, 748–759. https://doi.org/10.1038/s41565-021-00931-2.

79. Cecchin, R., Troyer, Z., Witwer, K., and Morris, K.V. (2023). Extracellular vesicles: The next generation in gene therapy delivery. Mol. Ther. *31*, 1225–1230. https://doi.org/10.1016/j.ymthe.2023.01.021.

80. Guo, S.C., Tao, S.C., Yin, W.J., Qi, X., Yuan, T., and Zhang, C.Q. (2017). Exosomes derived from platelet-rich plasma promote the re-epithelization of chronic cutaneous wounds via activation of YAP in a diabetic rat model. Theranostics *7*, 81–96. https://doi.org/10.7150/thno.16803.

81. Eming, S.A., Martin, P., and Tomic-Canic, M. (2014). Wound repair and regeneration: mechanisms, signaling, and translation. Sci. Transl. Med. *6*, 265sr6.

82. Huang, X., Liu, G., Guo, J., and Su, Z. (2018). The PI3K/AKT pathway in obesity and type 2 diabetes. Int. J. Biol. Sci. *14*, 1483–1496. https://doi.org/10.7150/ijbs.27173.

83. Wolf, G. (2000). Cell cycle regulation in diabetic nephropathy. Kidney Int. Suppl. *77*, S59–S66. https://doi.org/10.1046/j.1523-1755.2000.07710.x.

84. Deng, B., Song, A., and Zhang, C. (2023). Cell-Cycle Dysregulation in the Pathogenesis of Diabetic Kidney Disease: An Update. Int. J. Mol. Sci. *24*, 2133. https://doi.org/10.3390/ijms24032133.

85. Lorenzi, M., Nordberg, J.A., and Toledo, S. (1987). High glucose prolongs cell-cycle traversal of cultured human endothelial cells. Diabetes *36*, 1261–1267.

86. Murayama, K., Kimura, T., Tarutani, M., Tomooka, M., Hayashi, R., Okabe, M., Nishida, K., Itami, S., Katayama, I., and Nakano, T. (2007). Akt activation induces epidermal hyperplasia and proliferation of epidermal progenitors. Oncogene *26*, 4882–4888. https://doi.org/10.1038/sj.onc.1210274.

87. Goren, I., Müller, E., Schiefelbein, D., Gutwein, P., Seitz, O., Pfeilschifter, J., and Frank, S. (2009). Akt1 controls insulin-driven VEGF biosynthesis from keratinocytes: implications for normal and diabetes-impaired skin repair in mice. J. Invest. Dermatol. *129*, 752–764. https://doi.org/10.1038/jid.2008.230.

88. Cibrian, D., de la Fuente, H., and Sánchez-Madrid, F. (2020). Metabolic Pathways That Control Skin Homeostasis and Inflammation. Trends Mol. Med. *26*, 975–986. https://doi.org/10.1016/j.molmed.2020.04.004.

89. Piipponen, M., Li, D., and Landen, N.X. (2020). The Immune Functions of Keratinocytes in Skin Wound Healing. Int. J. Mol. Sci. *21*, 8790. https://doi.org/10.3390/ijms21228790.

90. Werner, S., Krieg, T., and Smola, H. (2007). Keratinocyte-fibroblast interactions in wound healing. J. Invest. Dermatol. *127*, 998–1008. https://doi.org/10.1038/sj.jid.5700786.

91. Gonzalez, A.C.d.O., Costa, T.F., Andrade, Z.d.A., and Medrado, A.R.A.P. (2016). Wound healing - A literature review. Bras. Dermatol. *91*, 614–620. https://doi.org/10.1590/abd1806-4841.20164741.

92. Ammann, K.R., DeCook, K.J., Li, M., and Slepian, M.J. (2019). Migration versus proliferation as contributor to *in vitro* wound healing of vascular endothelial and smooth muscle cells. Exp. Cell Res. *376*, 58–66. https://doi.org/10.1016/j.yexcr.2019.01.011.

93. Inaba, H., Yamakawa, D., Tomono, Y., Enomoto, A., Mii, S., Kasahara, K., Goto, H., and Inagaki, M. (2018). Regulation of keratin 5/14 intermediate filaments by CDK1, Aurora-B, and Rho-kinase. Biochem. Biophys. Res. Commun. *498*, 544–550. https://doi.org/10.1016/j.bbrc.2018.03.016.

94. Jones, M.C., Askari, J.A., Humphries, J.D., and Humphries, M.J. (2018). Cell adhesion is regulated by CDK1 during the cell cycle. J. Cell Biol. *217*, 3203–3218. https://doi.org/10.1083/jcb.201802088.

YMTHE, Volume *33*

Supplemental Information

CDK1-loaded extracellular vesicles promote cell cycle to reverse impaired wound healing in diabetic obese mice

Wooil Choi, Dong Jun Park, Robert A. Dorschner, Keita Nakatsutsumi, Michelle Yi, and Brian P. Eliceiri

Supplemental material

Table S1. List of materials

REAGENT or RESOURCE	SOURCE	IDENTIFIER
Antibodies		
anti-Alix mAb	Cell Signaling Technology	Cat# 92880; RRID: AB_2800192
anti-Calnexin mAb	Cell Signaling Technology	Cat# 2679; RRID: AB_2228381
anti-CD63 pAb	Thermo Fisher	Cat# PA5-92370; RRID: AB_2806456
anti-Cdc2 mAb	Cell Signaling Technology	Cat# 9116; RRID: AB_2074795
anti-CD81 mAb	Cell Signaling Technology	Cat# 56039; RRID: AB_2924772
anti-Ki-67 mAb	Thermo Fisher	Cat# MA5-14520; RRID: AB_10979488
anti-Akt mAb	Cell Signaling Technology	Cat# 4691; RRID: AB_915783
anti-pAkt (Ser473) mAb	Cell Signaling Technology	Cat# 4060; RRID: AB_2315049
anti-4E-BP1 mAb	Cell Signaling Technology	Cat# 9644; RRID: AB_2097841
anti-p4E-BP1 (Thr37/46) mAb	Cell Signaling Technology	Cat# 2855; RRID: AB_560835
anti-Erk1/2 mAb	Cell Signaling Technology	Cat# 4695; RRID: AB_390779
anti-pErk1/2 (Thr202/Thr204) mAb	Cell Signaling Technology	Cat# 4370; RRID: AB_2315112
anti-p27 Kip1 mAb	Cell Signaling Technology	Cat# 3686; RRID: AB_2077850
anti-pHistone H3 (Ser10) mAb	Cell Signaling Technology	Cat# 9706; RRID: AB_331748
anti-Acetyl-α-tubulin mAb	Cell Signaling Technology	Cat# 5335; RRID: AB_10544694
anti-β-actin mAb	Cell Signlaing Technology	Cat# 3700; RRID: AB_2242334
anti-Mouse IgG, HRP-linked	Cell Signaling Technology	Cat# 7076; RRID: AB_330924
anti-Rabbit IgG, HRP-linked	Cell Signaling Technology	Cat# 7074; RRID: AB_2099233
anti-Rabbit IgG-Alexa Fluor™ 488	Thermo Fisher	Cat# A11008; RRID: AB_143165
anti-Rabbit IgG-Alexa Fluor™ 546	Thermo Fisher	Cat# A11010; RRID: AB_2534077
anti-Mouse IgG-Alexa Fluor™ 488	Thermo Fisher	Cat# A11029; RRID: AB_2534088
anti-Mouse IgG-Alexa Fluor™ 546	Thermo Fisher	Cat# A11030; RRID: AB_2737024
anti-hCD9 Ab	Cellarcus Bioscience Inc.	Cat# CBS10-PE
anti-hCD63 Ab	Cellarcus Bioscience Inc.	Cat# CBS11-PE
anti-hCD81 Ab	Cellarcus Bioscience Inc.	Cat# CBS12-PE
Isotype IgG1, κ control	Cellarcus Bioscience Inc.	Cat# CBS29-PE
Chemicals, Enzymes		
DMEM-high glucose medium	Thermo Fisher	Cat# 12430054
Fetal Bovine Serum (FBS)	Sigma Aldrich	Cat# F0926
Antibiotic-Antimycotic (100X)	Thermo Fisher	Cat# 15240062
0.4% Trypan blue	Thermo Fisher	Cat# T10282
Xho I	New England Biolabs	Cat# R0146
Not I	New England Biolabs	Cat# R1089S
Lipofectamine™ 2000	Thermo Fisher	Cat# L3000150

Opti-MEM™	Thermo Fisher	Cat# 31985062
Bovine exosome-delpleted FBS	System Biosciences	Cat# EXO-FBS-50A-1
ExoQuick-TC	System Biosciences	Cat# EXOTC-50A-1
Pierce™ RIPA buffer	Thermo Fisher	Cat# 89901
Halt™ Protease & phosphatase inhibitor cocktail	Thermo Fisher	Cat# 78442
Pierce™ Bovine serum albumin	Thermo Fisher	Cat# 23209
NuPAGE™ LDS sample buffer (4X)	Thermo Fisher	Cat# NP008
Dithiothreitol (DTT)	Thermo Fisher	Cat# 15508013
Non-fat dry milk	Cell Signaling Technology	Cat# 9999
SignalFire™ Elie ECL reagent	Cell Signaling Technology	Cat# 12757
vCal nanoRainbow beads	Cellarcus Bioscience Inc.	Cat# CBS6
Mitomycin C	Sigma-Aldrich	Cat# M0440
Tissue-Tek® O.C.T. Compound	Sakura	Cat# 4583
Paraformaldehyde aqueous solution	Electron Microscopy Science	Cat# 15710
Triton X 100		
Fluoroshield™ with DAPI	Sigma-Aldrich	Cat# F6057
LysoTracker™ Red DND-99	Thermo Fisher	Cat# L7528
1X Phosphate-buffered saline	Thermo Fisher	Cat# 10010023
Tris-buffered saline with Tween 20	Cell Signaling Technology	Cat# 9997
MACSQuant running buffer	Miltenyi Biotec	Cat# 130-092-747
Critical Commercial Assay		
ZymoPURE II plasmid maxiprep kit	Zymo Research	Cat# D4203
Pierce™ BCA protein assay	Thermo Fisher	Cat# 23225
Pierce™ Reversibl protein stain kit	Thermo Fisher	Cat# 24585
vFC™ Vesicle flow cytometry assay	Cellarcus Biosciences Inc.	Cat# CBS4HP
Cell counting kit-8 assay	Dojindo	Cat# CK04
Cell cycle assay solution deep red	Dojindo	Cat# C548
Recombinant DNA		
XPack CMV-XP-MCS	System Biosciences	Cat# XPAK510PA-1
XPack CMV-XP-GFP	System Biosciences	Cat# XPAK530CL-1
Human CDK1 untagged clone	Origene	Cat# SC111605
Experimental models: Animals		
B6.VJS(D)-Lepr$^{db/db}$/J mice	Jackson lab	JAX000697; RRID: IMSR_JAX:000697
Experimental models: Cell lines		
HEK293T	Takara Bio	Cat# 632180; RRID: CVCL_4401
HaCaT		
Experimental models: Bacterial strain		
NEB stable competent *E. coli* (K12)	New England Biolabs	Cat# C3040I

Software and Equipment		
GraphPad Prism10 software	GraphPad Software	https://www.graphpad.com/
FCS express	De Novo	https://denovosoftware.com/
Aperio ImageScope (v12.4.6)	Leica Biosystem	https://www.leicabiosystems.com/digital-pathology/manage/aperio-imagescope/
ZEN blue (v3.4.91)	Carl Zeiss Microscopy GmbH	https://www.zeiss.com/microscopy/en/products/software/zeiss-zen.html
FlowJo software (v10.8.2)	FlowJo™, LLC	https://www.flowjo.com/
Image J (v1.54i)	NIH	https://imagej.nih.gov/ij/download.html
CytoFLEX Flow Cytometer	Beckman Coulter	https://www.beckman.com/flow-cytometry/research-flow-cytometers/cytoflex
Xenogen IVIS-Lumina	Caliper life science Inc.	http://www.caliperls.com
MACSQuant Analyzer 10	Miltenyi Biotec	https://www.miltenyibiotec.com/US-en/products/macsquant-analyzer-10.html
Retiga R6 CCD camera	Teledyne photometrics	https://www.photometrics.com/products/qimaging-ccd-family/retiga-r6
AXR confocal microscopy	Nikon	http://www.microscope.healthcare.nikon.com
Others		
12% Bis-tris mini gel	Thermo Fisher	Cat# NP0342BOX
PVDF membrane	Thermo Fisher	Cat# LC2005
96-well v bottom plate	Sarstedt	Cat# NC0068972
Oasis Nylon Suture	Med Vet International	Cat# MV-662
Betadine (10% povidone-iodine solution)	Thermo Fisher	Cat# NDC 67618-150-01
4 mm punch	Acuderm Inc.	Cat# P450
Silicone ring	Crace Bio-Labs	Cat# GBLRD476687
Surgical glue	Thermo Fisher	Cat# 1469SB
Tegaderm™ transparent dressing film	3M	Cat# 1622w
2-well silicon insert	Ibidi	Cat# 80209
Black 12-well plate with glass like bottom	Cellvis	Cat# P12-1.5P

Figure S1

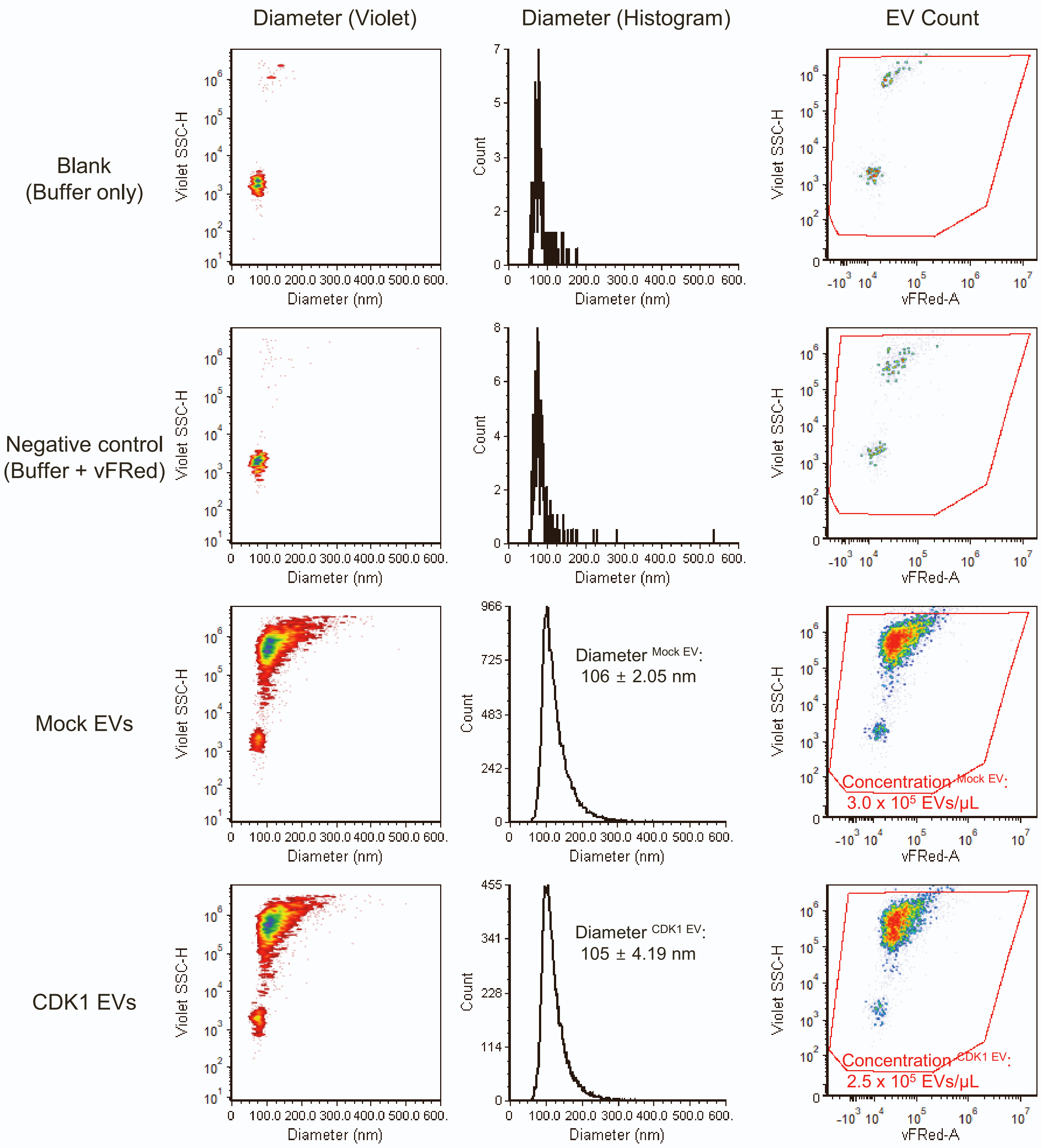

Figure S1. Controls for single vesicle flow cytometry (vFC) based on concentration of samples by polymeric precipitation. Representative controls showing event distribution in a running buffer only (Blank), negative control that has running buffer with fluorescent lipophilic membrane dye vFRed without sample, Mock sEVs and CDK1-loaded sEVs with vFRed dye. The left column contains plots of size based on calibration with Nanorainbow beads vs Violet SSC-H. The middle column shows diameter vs. count to obtain a size distribution and further supporting the ~110 nm population. The right column is a distribution of vFRed positive events vs. Violet SSC-H with concentration calculated based on events, rate, and volume.

Figure S2

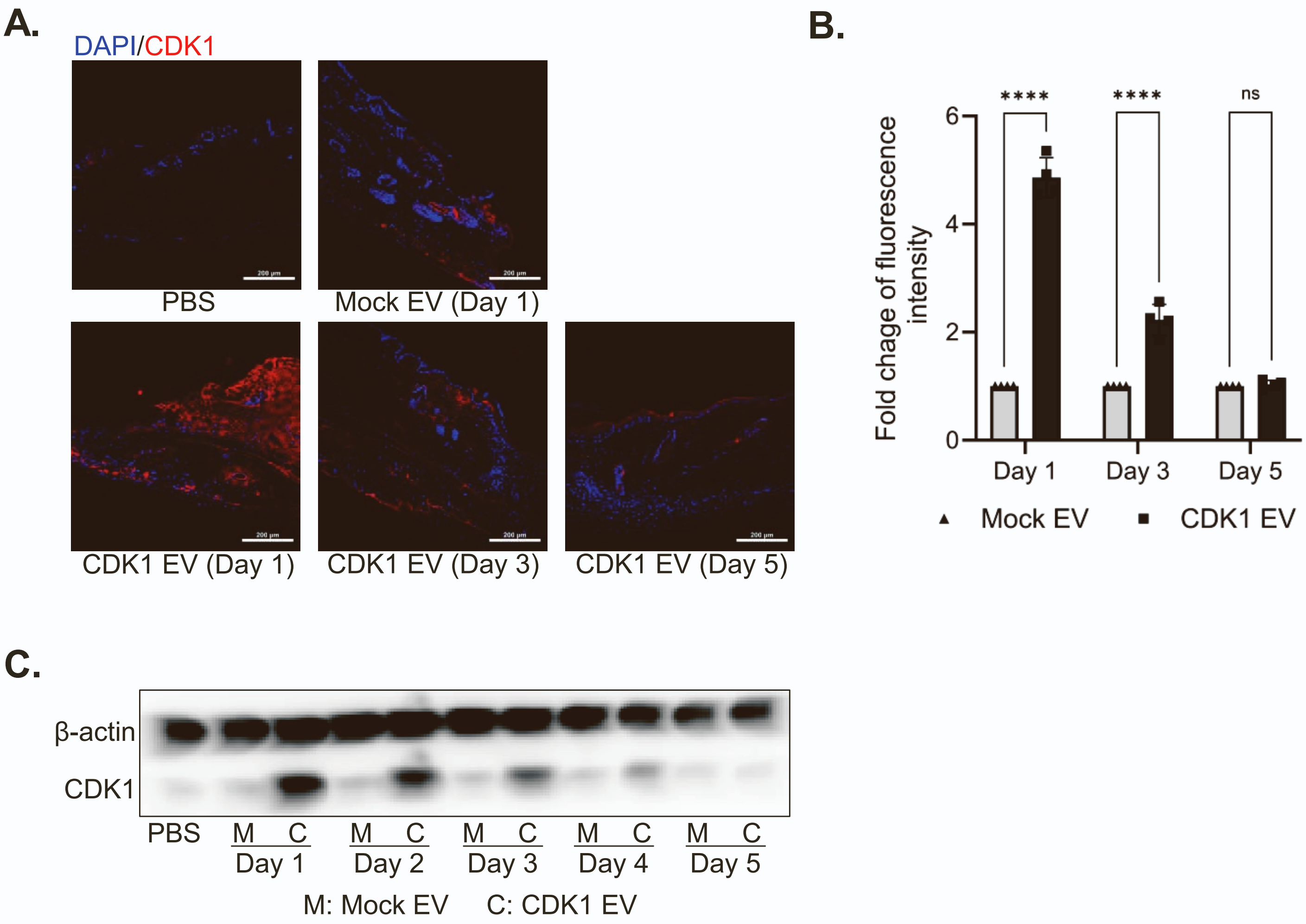

Figure S2. Retention time of CDK1-loaded sEVs in wound area. (A) Representative immunofluorescent images of wound margin with an anti-CDK1 antibody (red), and counter-stained with a nuclear stain (blue) post-sEV treatment (Scale bar: 200 μm) and (B) their quantification (n = 4, $^{****}p < 0.0001$). (C) Immunoblotting of CDK1 in wound area.

Figure S3

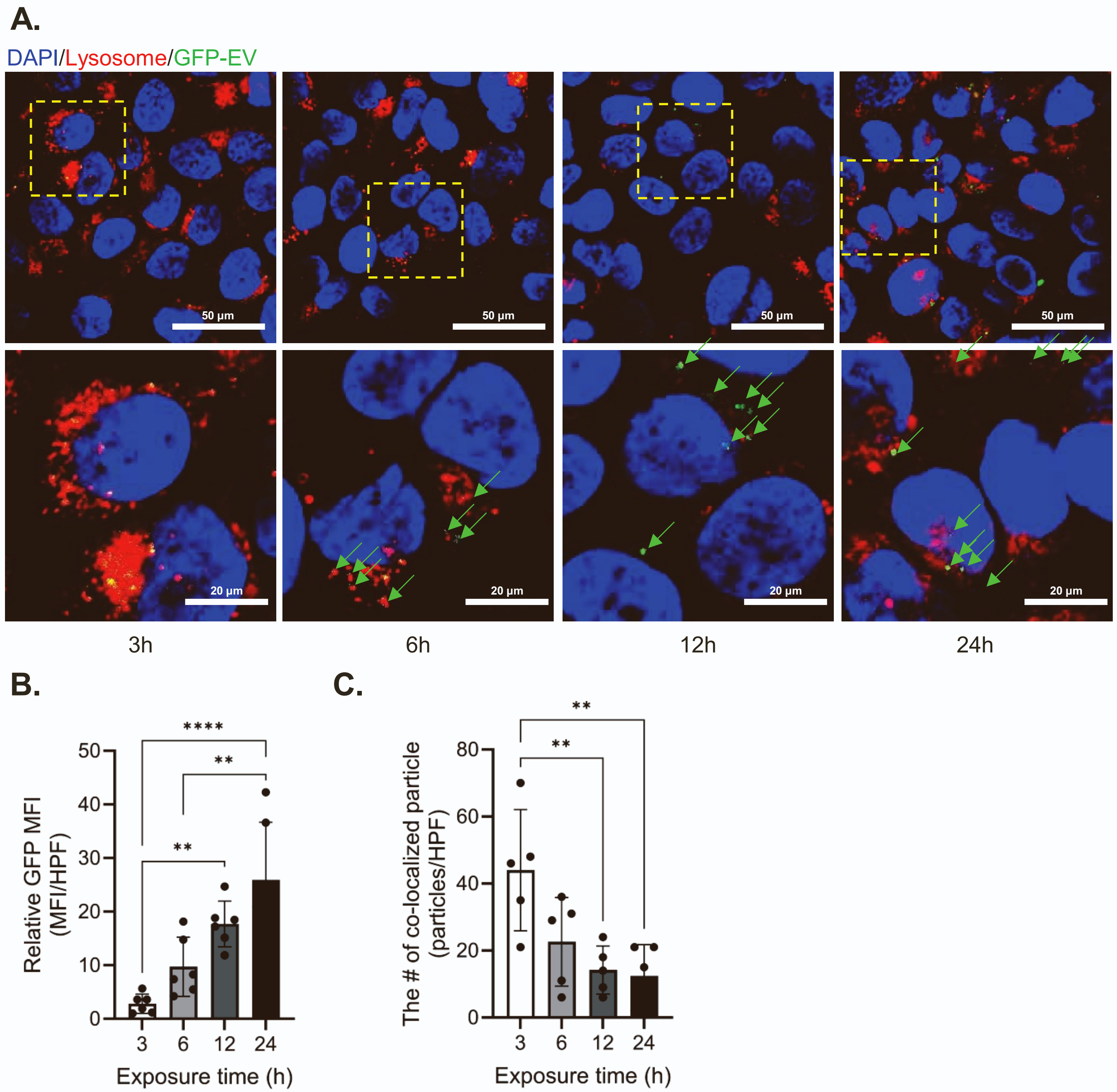

Figure S3. EV cargo release occurs from lysosomes. (A) Immunofluorescent images stained with lysotracker (red) incubated with GFP-loaded sEVs (green) for 3, 6, 12, and 24 hours. Yellow particles represent co-localization of lysosome and GFP and green arrows point escaped GFP (Scale bar: 50 µm). (B) quantified fluorescence intensity of GFP for 24 hours ($n = 6$, $^{****}p < 0.0001$, $^{**}p < 0.01$). (C) quantification of co-localization of lysosome and GFP ($n = 6$, $^{**}p < 0.01$).

Figure S4

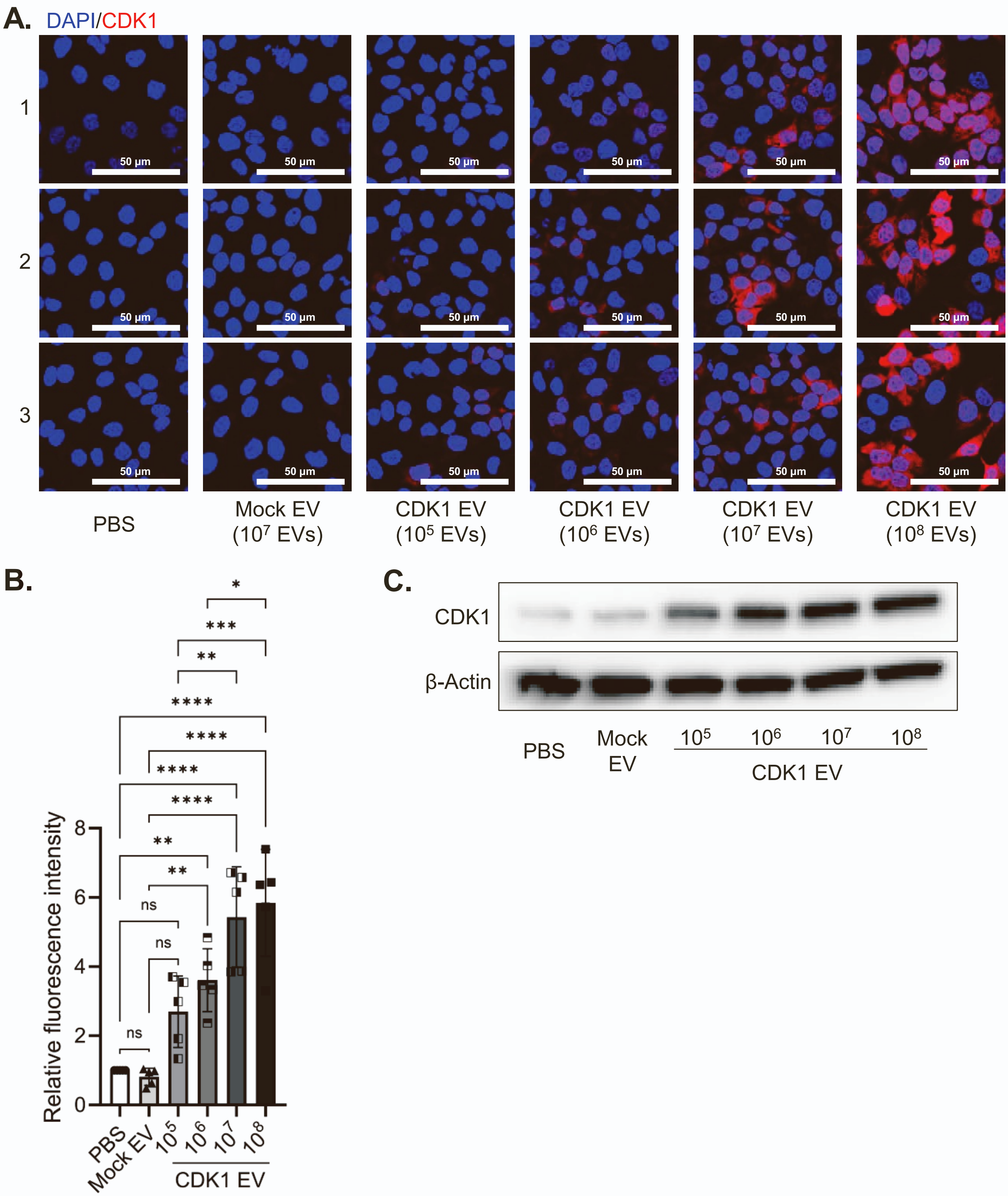

Figure S4. Release kinetics of CDK1 in recipient keratinocytes from sEVs. (A) immunofluorescent images stained with CDK1 (red), and counter stained with DAPI for nuclei (blue) after CDK1-loaded sEV treatment (Scale bar: 50 μm), and their quantification (B), ($n = 5$, $^{****}p < 0.0001$, $^{***}p < 0.001$, $^{**}p < 0.01$, $^{*}p < 0.05$). (C) immunoblot of CDK1 in recipient whole cell lysate after CDK1-loaded sEV treatment.

Figure S5

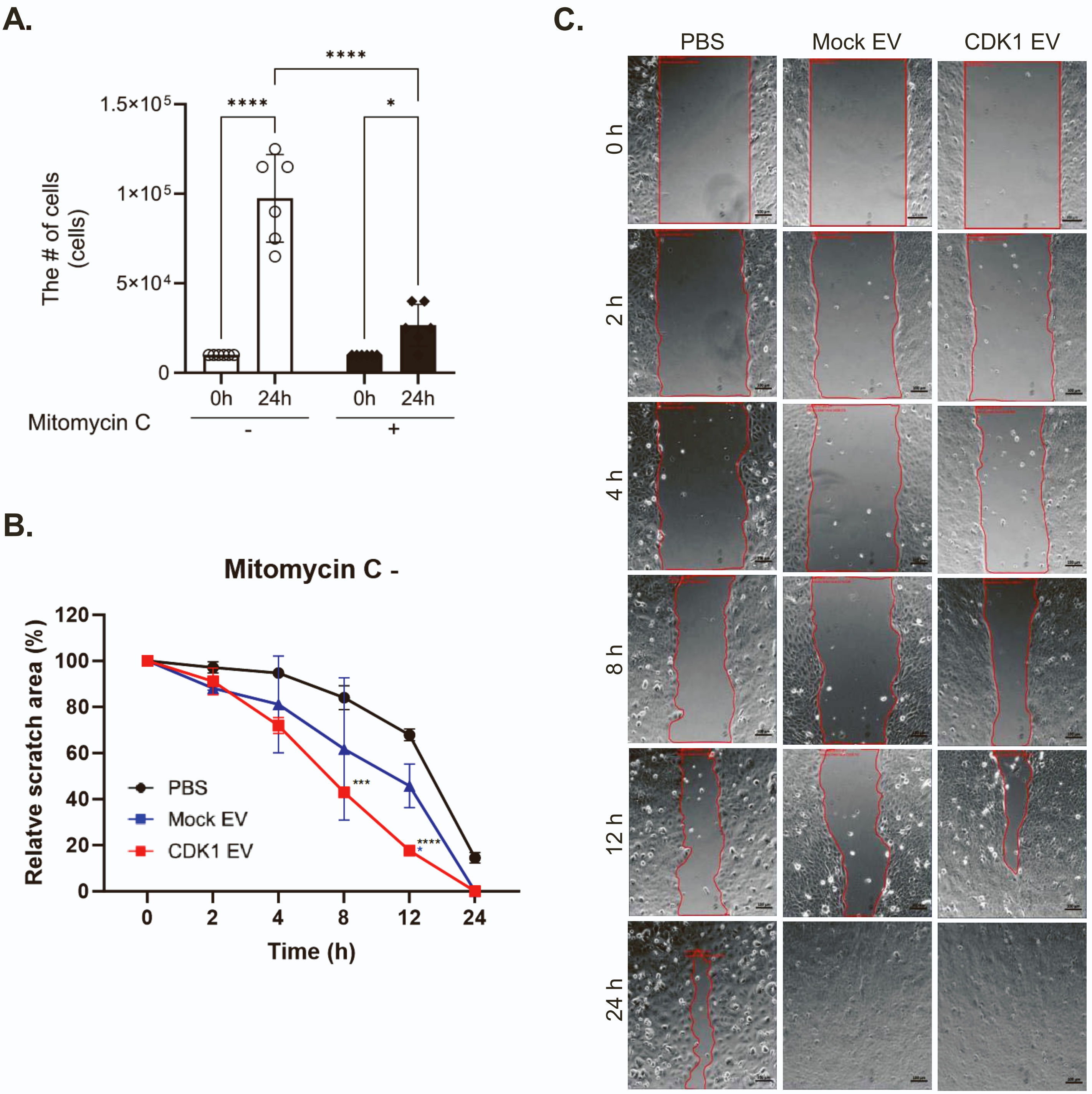

Figure S5. In vitro migration assay by CDK1 delivered to human keratinocyte by sEV treatment. (A) Proliferation inhibitory efficacy of mitomycin C in human keratinocyte (n = 6, $^{****}p < 0.0001$). (B) quantification of cell migration without proliferation arrest by mitomycin C, and their representative images (C), (Scale bar: 100 µm, $^{****}p < 0.0001$, $^{***}p < 0.001$, $^{*}p < 0.05$).

Figure S6

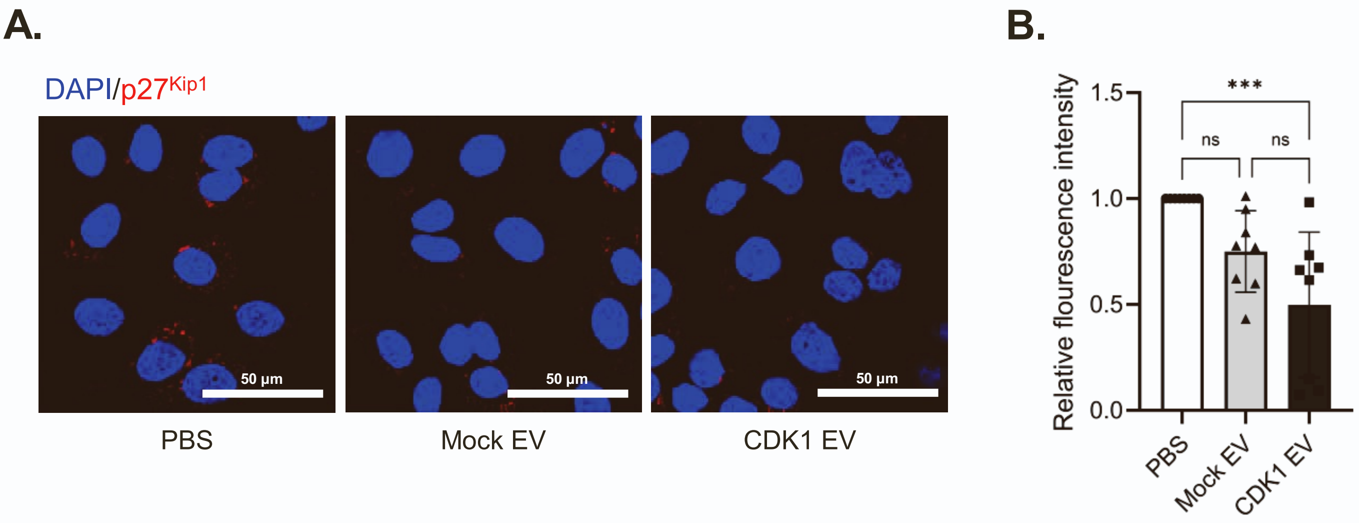

Figure S6. Repression of p27^{Kip1} by activated AKT and ERK signaling. (A) immunofluorescent images stained with p27^{Kip1} (red), and counter stained with DAPI for nuclei (blue) after CDK1-loaded sEV treatment, and their quantification (B), (Scale bar: 50 μm, n = 8, $^{***}p < 0.001$).
